# GWAS analysis to elucidate genetic composition underlying a photoperiod-insensitive rice population, North Korea

**DOI:** 10.3389/fgene.2022.1036747

**Published:** 2022-12-07

**Authors:** Chuluuntsetseg Jadamba, Richie L. Vea, Jung-Hoon Ryu, Nam-Chon Paek, Su Jang, Joong Hyoun Chin, Soo-Cheul Yoo

**Affiliations:** ^1^ Crop Molecular Breeding Laboratory, Department of Plant Life and Environmental Science, Hankyong National University, Anseong, South Korea; ^2^ Bureau of Plant Industry, National Seed Quality Control Services, San Mateo, Isabela Philippines; ^3^ Department of Plant Science, Plant Genomics and Breeding Institute, Research Institute of Agriculture and Life Sciences, Seoul National University, Seoul, South Korea; ^4^ Department of Integrative Biological Sciences and Industry, Sejong University, Seoul, South Korea; ^5^ Carbon-Neutral Resources Research Center, Hankyong National University, Seoul, South Korea

**Keywords:** GWAS, photoperiod sensitivity, heading date, North Korean rice, haplotyping, regional adaptation

## Abstract

Heading date (Hd) is one of the main factors determining rice production and regional adaptation. To identify the genetic factors involved in the wide regional adaptability of rice, we conducted a genome-wide association study (GWAS) with 190 North Korean rice accessions selected for non-precocious flowering in the Philippines, a low-latitude region. Using both linear mixed models (LMM) and fixed and random model circulating probability unification (FarmCPU), we identified five significant loci for Hd in trials in 2018 and 2019. Among the five lead single nucleotide polymorphisms (SNPs), three were located adjacent to the known Hd genes, Heading date 3a (*Hd3a*), Heading date 5 (*Hd5*), and *GF14-c.* In contrast, three SNPs were located in novel loci with minor effects on heading. Further GWAS analysis for photoperiod insensitivity (PS) revealed no significant genes associated with PS, supporting that this North Korean (NK) population is largely photoperiod-insensitive. Haplotyping analysis showed that more than 80% of the NK varieties harbored nonfunctional alleles of major Hd genes investigated, of which a nonfunctional allele of *Heading date 1* (*Hd1*) was observed in 66% of the varieties. Geographical distribution analysis of Hd allele combination types showed that nonfunctional alleles of floral repressor Hd genes enabled rice cultivation in high-latitude regions. In contrast, *Hd1* alleles largely contributed to the wide regional adaptation of rice varieties. In conclusion, an allelic combination of Hd genes is critical for rice cultivation across wide areas.

## Introduction

Rice (*Oryza sativa* L.)*,* a major cereal crop, especially in Asia, consists of two subspecies: *indica* and *japonica*. As a facultative short-day (SD) plant, rice flowering can be promoted under SD conditions and repressed under long-day (LD) conditions (Li et al., 2020). Heading date (Hd), which involves the transition from vegetative to reproductive growth, is the most critical growth stage of rice and affects rice cultivation over a wide range of latitudes and the regional adaptability of rice plants (Izawa, 2007; Koo et al., 2013). Photoperiod sensitivity (PS) is the plant’s response to the length of the day, and its Hd can be determined by its vegetative phase (Yano et al., 2000; Fujino, 2003). The photoperiodic control of flowering is one of the most significant components of the interaction between plants and their environment, and many genes are involved in this photoperiodic response (Nayyeripasand et al., 2013).

*Hd1* promotes heading under SD conditions and represses heading under LD conditions by regulating the expression of *Hd3a*, a florigen gene in rice (Yano et al., 2000; Kojima et al., 2002; Tamaki et al., 2007). *Hd3a*, which was identified as a quantitative trait locus (QTL) for flowering time, is a key activator of flowering in rice (Kojima et al., 2002). Many studies have suggested that *FT/Hd3a* represents a florigen-type mobile flowering signal (Corbesier et al., 2007; Jaeger and Wigge, 2007; Mathieu et al., 2007; Lin et al., 2000; Kwon et al., 2014). *Hd3a* expression is regulated by *Hd1* and Early heading 1 (*Ehd1*), a B-type response regulator that functions independently of *Hd1* (Yano et al., 2000; Hayama et al., 2003; Doi et al., 2004). The *Hd3a* gene acts as a flowering signal under SD conditions (Komiya et al., 2008; Nayyeripasand et al., 2013), and its expression can be regulated by *OsPRR37* (*Hd2*) to suppress flowering under LD conditions (Koo et al., 2013). Heading date 5 (*Hd5*) delays the heading date under both LD and SD conditions (Wei et al., 2010). *Hd5*, which encodes a putative HAP3 subunit of the HAP complex, controls rice photoperiod sensitivity by forming a complex that interacts with *Ghd7*. *Hd5* was identified as a major effect locus affecting flowering, with the dual function of inhibiting flowering under LD conditions and promoting flowering under SD conditions by regulating *Ehd1* and *Hd3a* (Wang et al., 2019).

Genome-wide association study (GWAS) has been widely used to identify single nucleotide polymorphisms (SNPs) that are significantly associated with various important agronomic traits in rice (Huang et al., 2011; Zhao et al., 2011). GWAS is an alternative mapping strategy that utilizes genetically diverse populations of unrelated individuals (Famoso et al., 2011). Hd is one of the traits extensively studied by GWAS in rice (Huang et al., 2011; Zhao et al., 2020). *Hd3a* and *OsGI* were detected in both *indica* and *japonica* populations, with 32 new signals associated with flowering time loci in 950 rice varieties worldwide (Huang et al., 2011). *OsGI* has also been identified by GWAS analysis in the African rice population for both early and late sowing (Cubry et al., 2020). A worldwide collection of 529 *Oryza sativa* accessions was analyzed by GWAS and identified three CCT genes (*OsPRR59*, *OsP, and OsCMF10*) and two MADS genes (*OsMADS87* and *OsMADS30*) (Liu et al., 2021) for the Hd phenotype. Additionally, 69 accessions from the world rice core collection were analyzed using GWAS, and candidate genes associated with Hd and seed characteristics were detected (Tanaka et al., 2020).

The North Korean (NK) rice collection is a unique source of breeding materials since the NK rice collection has not been extensively utilized for genetic analysis. Thus, revealing the genetic basis of the NK rice collection would be beneficial for discovering new genetic factors associated with Hd and understanding its genetic composition. This study used 190 NK rice varieties selected from the NK rice collection for non-precocious flowering and normal growth in the low-latitude Philippines, indicating that these varieties are mainly photoperiod-insensitive (PIS). Here, we performed GWAS and haplotype analyses using the selected NK rice population and proposed significant genetic loci associated with Hd regulation and the genetic composition of this population by analyzing the allelic combination of the major Hd genes. The geographical distribution of these Hd allelic combinations was also discussed with respect to regional adaptation.

## Materials and methods

### Plant material and phenotype analysis

We used 190 NK rice cultivars selected for non-precocious flowering and normal growing from 4000 NK rice varieties in the experimental field of the International Rice Research Institute (IRRI), Los Baños, Philippines (14°35 N, 120°58 E) in 2010. *Japonica* rice varieties are typically grown in temperate countries with long photoperiods. The Democratic People’s Republic of Korea (DPRK or North Korea) (40.3399°N, 127.5101°E) is a country that cultivates *japonica* rice. NK rice accessions were deposited at the IRRI for germplasm conservation and domestication. The rice varieties were planted in the experimental area of Hankyong National University, Anseong-si, South Korea (37°0N, 127°16E), under natural LD conditions. The sowing date of the rice cultivars was 30 April 2018, and 2019 in Anseong Si, South Korea, and they were transplanted on 09 June 2018, and 24 May 2019. The average day length in South Korea was approximately 13.5 h, and the maximum temperature range was 27–39°C in Anseong-si (Supplementary Figure S1) Hd was recorded as the number of days from sowing to the day when the spikelet of the first panicle emerged, based on visual observation of each rice variety. The data for natural SD conditions were adapted from the IRRI; the emergence date was April 23 and 12 May 2010, and they were transplanted on May 29 and 9 June 2010, respectively. The maximum temperature ranged from 32 to 36°C, Supplementary Figure S1) (World weather online 2018) average day length is 12 h (short day). The PS data of each entry were calculated from the difference in Hd between the SD and LD (Wei et al., 2010). We used an additional reference of 25 rice accessions from the 3,000 Rice Genomes Project (3 K-RGP) for clear subspecies classification (*TemJ, TroJ, Ind, Aro, Aus*) (Supplementary Table S1). 3 K-RGP (additional 25 variety) data were only used for the NJ tree and PC analysis.

### NGS analysis and genotyping

Genomic DNA was extracted from 190 NK rice varieties using the CTAB method (Murray and Thompson, 1980). Young leaves were collected with beads in a 2 ml tube and homogenized after freezing in liquid nitrogen and stored at -70°C until use in NGS. The DNA quality was analyzed using a UV spectrophotometer and the Gen5 2.07 program. The gDNA (1 mg) was sheared using an S220 ultrasonicator (Covaris). Library preparation was performed using the MGIEasy DNA library prep kit (MGI) according to the manufacturer’s instructions. Briefly, after the size selection of fragmented gDNA using AMPure XP magnetic beads, the fragmented gDNA was end-repaired and A-tailed at 37°C for 30°min and 65°C for 15 min. The indexing adapter was ligated to the ends of the DNA fragments at 23°C for 60 min. After cleanup of the adapter-ligated DNA, PCR was performed to enrich the DNA fragments with adapter molecules. Thermocycler conditions were as follows: 95°C for 3°min; seven cycles of 98°C for 20°s, 60°C for 15 s, and 72°C for 30°s; and a final extension at 72°C for 10 min. The double-stranded library was quantified using a QuantiFluor ONE dsDNA System (Promega). The library was circularized at 37°C for 30 min and then digested at 37°C for 30°min, followed by a cleanup of the circularization product. The library was incubated at 30°C for 25 min using the DNB enzyme to prepare a DNA nanoball (DNB). Finally, the library was quantified using the QuantiFluor ssDNA system (Promega). Sequencing of the prepared DNB was conducted using the MGI seq system (MGI) with 150 bp paired-end reads.

### Genome-wide association mapping

GWAS analysis was performed using mixed linear models (MLM) (Zhang et al., 2010) and the fixed and random model circulating probability unification (FarmCPU) model implemented in GAPIT and rMVP (memory-efficient, visualization-enhanced, and parallel-accelerated R package) packages in R, respectively (Lipka et al., 2012). In our study, a total of 1,048,576 SNPs with minor allele frequency ≥0.05, missing data rate of ≤0.25, minimum genotype quality ≥30, and minimum depth ≥4 filtered by PLINK, were used for association analysis. Linkage disequilibrium (LD)-based SNP pruning was performed using PLINK v1.9 with the command (--Indep-pairwise “50 5 0.2”). Non-independent SNPs were removed, and 82,663 effective and independent SNPs remained. False positives in the GWAS were corrected using the Bonferroni test. Using the Bonferroni multiple test correction (0.05/number of independent SNP; at a 5% level of significance), the calculated threshold value was set as - log_10_ (*p*) = 6.05.

### Population structure, linkage disequilibrium, and haplotype

Neighbor-joining (NJ) trees were constructed to infer genetic relationships using MEGA v7 (Kumar et al., 2018). LD analysis identified 100 kb–1 Mb around the lead SNPs as candidate genomic regions for gene identification. LD patterns between lead SNPs and other SNPs were evaluated using PLINK v1.9 with the *R* ^2^ command to calculate pairwise genotype correlations (*R* ^2^) (Purcell et al., 2007). Lead SNPs were defined as SNPs with the lowest *p*-value in the loci, including significant SNPs. Haplotype analysis was performed using raw, unfiltered genotype data to access all variants of the SNP and InDel calls. After the removal of heterozygotes and missing data, SNPs and InDels in the exon regions were used for LD and haplotype variation analyses. The target genomic regions for haplotyping were generated using the site-filtering options of the variant call format (vcf) (--from-bp and --to-bp). We scanned the genomic regions of the significant loci of the genomic pseudomolecules of *japonica cv*. Nipponbare from the Michigan State University (MSU) Rice Database using Annotation version 6.1. Among these genes, candidate genes were identified based on the LD and predicted function of genes in relation to Hd.

## Result

### Population structure of North Korean rice accessions

**FIGURE 1:**
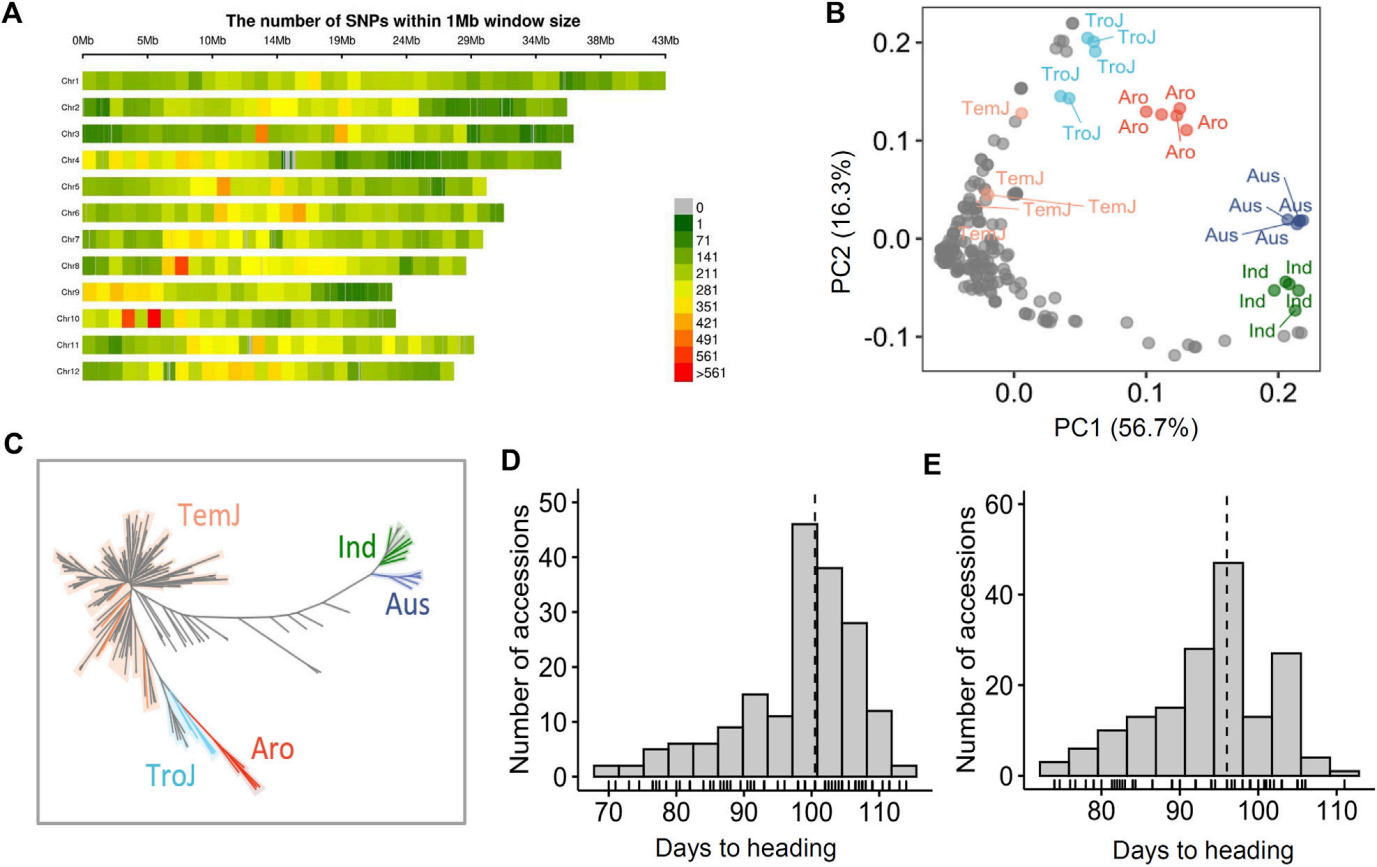
Population structure and phenotypic variation in North Korean rice accessions. **(A)** Chromosome-wise SNP density plot representing the number of SNPs within a 1 Mb window size. **(B)** PCA based on 215 varieties (190 NK and 3K-RGP derived 25 rice accessions). The two top principal components (PC1 and PC2) explained 73% of the genetic variation. Accessions are color-coded: orange, *TemJ*; light blue, *TroJ*; red, *Aro*; dark blue, *Aus*; green, *Ind* **(C)** Neighbor-joining tree. **(D and E)** Histograms showing phenotype distribution of heading date in 2018 **(D)** and 2019 **(E)** populations.

A total of 190 NK rice varieties were genotyped by NGS analysis, resulting in 1,048,576 high-quality SNPs. In total, 82,663 LD-pruned independent SNPs with a minor allele frequency (MAF) ≥ 0.05 were obtained for analysis of population structure. An additional 25 reference varieties consisting of various subspecies were selected from the 3 K-RGP database (Li et al., 2014). Their SNP data were used in the population structure analysis for clear subspecies classification. SNPs were uniformly distributed, covering the entire genome of the 12 chromosomes. Chromosome 10 possessed the highest SNP density, whereas chromosome one possessed the lowest SNP density (Figure 1A). Principal component analysis (PCA) was performed based on 82663 LD-pruned SNPs. Based on the PCA results, five subgroups [*Temperate Japonica* (*TemJ*), *Tropical Japonica* (*TroJ*), *Aromatic* (*Aro*), *Aus,* and *Indica* (*Ind*)] were clearly distinguished. PC1 and PC2 explained 56.7% and 16.3% of the total variance, respectively. The NJ tree analysis of genetic distance exhibited similar results, with most of the varieties, clearly clustered into one major *TemJ* group, and separated from the other subspecies, including *TroJ, Ind, Aus,* and *Aro* (Figure 1C). The NK population was clustered into *TemJ* (179), *TroJ* (5)*,* and *Ind* 6) subspecies, but not into *Aro* and *Aus*. Phenotype frequencies of the NK varieties displayed an approximately normal distribution for Hd (74–110°days in AS18 and 75–115°days in AS19) with a positive skewness (Figures 1D,E) and a similar distribution (48–112°days in the Philippines) (Supplementary Figure S2). Additionally, one-way ANOVA revealed significant differences in the Hd trait for genotypes (Supplementary Table S2), indicating that diverse phenotypic variation in this NK population may provide the opportunity to discover candidate genes for the Hd trait *via* genetic analysis (Ma et al., 2016).

### Genome-wide association mapping of Hd phenotype

**FIGURE 2:**
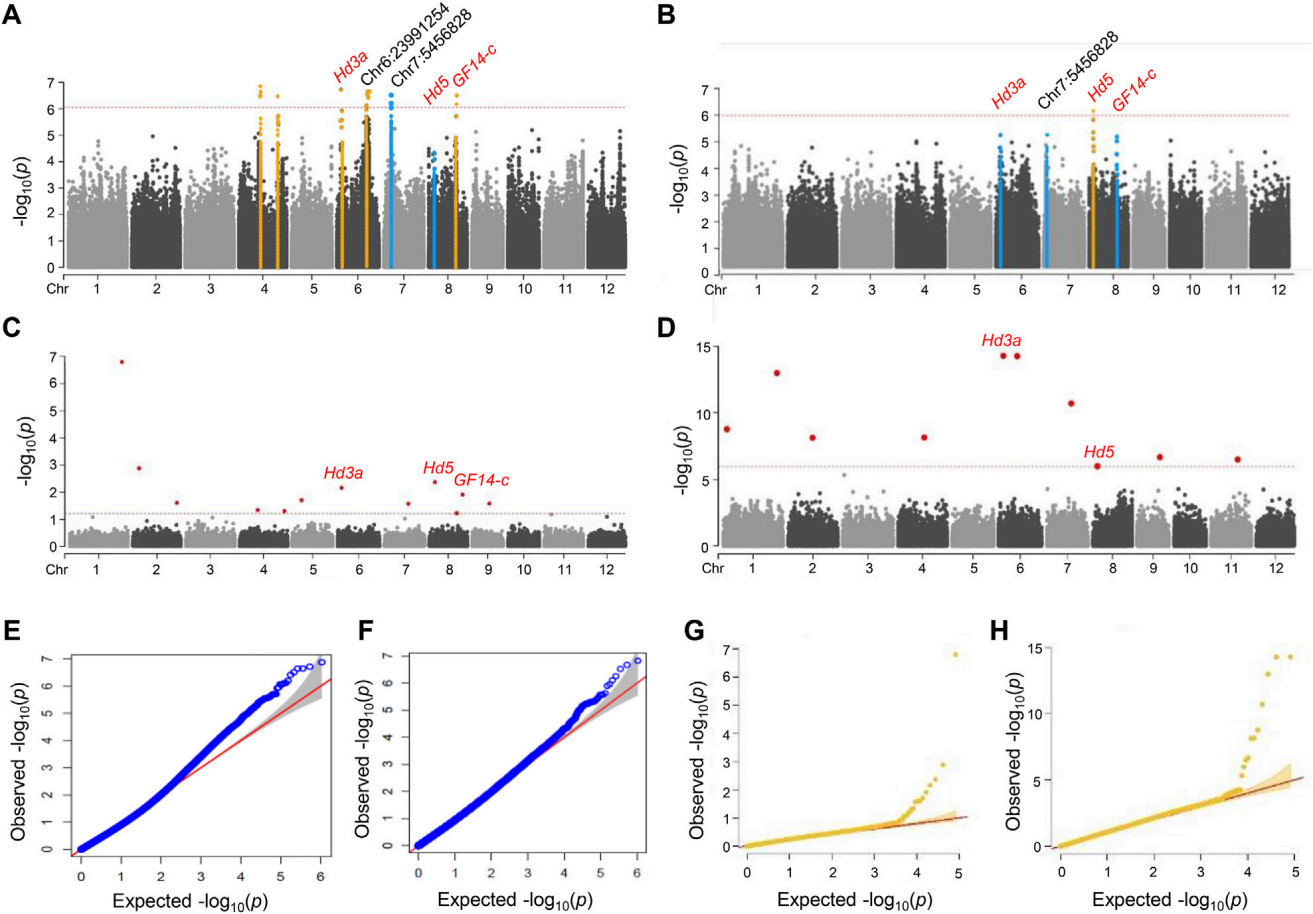
Manhattan and quantile-quantile plots for a GWAS of Hd phenotype in NK rice. **(A–D)** Manhattan plots of 2018 **(A)** and 2019 **(B)** in MLM model with GAPIT, and 2018. **(C)** and 2019. **(D)** in FarmCPU model with rMVP, respectively. Orange lines indicated significant SNPs correlated with Hd. Blue lines indicated major peaks not significantly correlated with Hd. Genome-wide significance threshold lines in MLM (*p* < 4.32 × 10^–7^) and FarmCPU model (*p* < 6.05 × 10^–7^) were shown as red dashed lines. Known and novel candidate genes are marked above lead SNPs. **(E–H)** Q-Q plot of 2018 **(E)** and 2019 **(F)** in MLM model with GAPIT and 2018 **(G)** and 2019. **(H)** in FarmCPU model with rMVP. For the Q-Q plots, the horizontal axis represents the expected -log_10_ (*p*), and the vertical axis is observed -log_10_ (*p*) of each SNP. The SNPs that had *p*-values deviated from the linear indicate reasonable positives.

To identify the genomic regions associated with the rice heading date phenotype, we performed a GWAS on 190 NK rice accessions. GWAS was performed using MLM (Figures 2A,B) and fixed and FarmCPU (Figures 2C,D) v2.07 models. A total of 10,994,201 SNPs were identified and only 1,048,576 SNPs that had the high quality with MAF ≥0.05 were included in the GWAS. The filtered SNPs were used for GWAS with phenotypic data. The heading date under natural LD conditions was recorded during the summer of 2018 and 2019 in Anseong, South Korea.

We identified five significant loci for the Hd phenotype in NK rice populations grown in 2018 by GWAS analysis using the MLM model. These lead SNPs are located on chromosomes 4, 6, and eight; Chr4:15932637 (*p* = 3.19 × 10^−7^), Chr4:29201109 (*p* = 2.26 × 10^−7^), Chr6:2971050 (*p* = 1.88 × 10^−7^), Chr6:23991254 (*p* = 2.20 × 10^−7^), and Chr8:20407302 (*p* = 1.93 × 10^−6^). LD analysis was performed to determine candidate genomic regions for each peak. Of these, two loci were co-localized with the known Hd genes, *Hd3a* and *GF14-c*, and no known Hd gene was associated with the other three lead SNPs (Figure 2; Table 1). The details of these significant association signals are presented in Supplementary Table S3. SNP Chr6:2971050, the most significant SNP in 2018, was located close to the *Hd3a*/*RFT1* gene (LOC_Os03g06320) (Figure 2A). This peak was also identified in the 2019 trial, although it was below the significance level (Figure 2B).

**TABLE 1:** Significant SNPs associated with Hd and PS.

Trait	Chr	Lead SNP	p-value	Candidate gene	Gene id	Description
HD	6	Chr6:2971050	1.87 × 10-7	*Hd3a/Rft1*	LOC_Os06g06320	Flowering Locus T gene
HD	6	Chr6:23991254	2.20 × 10-7	-	LOC_Os06g37840.1	Resistance protein
				-	LOC_Os06g37790	Expressed protein
HD	7	Chr7:5456828	3.02 × 10-7	-	LOC_Os07g10110.1	Expressed protein
HD	8	Chr8:20407302	1.93 × 10-6	*GF14-c*	LOC_Os08g33370	14-3-3 protein, putative
HD	8	Chr8:4688718	4.91 × 10-5	*Hd5*	LOC_Os08g07740	HAP3-like transcriptional-activator
PS	3	Chr3:10130737	5.45 × 10-7	*OsPhyB*	LOC_Os03g19590.1	phytochrome B, putative
PS	7	Chr7:25296269	7.87 × 10-6	*OsMADS18*	LOC_Os07g41370.1	OsMADS18 - MADS-box family gene

We also performed the FarmCPU models using rMVP for improved GWAS computation. FarmCPU and MLM were used iteratively to increase the statistical power of GWAS for detecting genetic loci associated with Hd. FarmCPU has higher statistical power than MLM for evaluating populations with either weak or strong population structures (Yin et al., 2021). With the FarmCPU model, we detected seven association signals (*p* < 6.05 × 10^–7^) for the 2018 and ten for 2019 trials (Figures 2E,F) (Supplementary Table S3). Three significant peaks were commonly detected in the 2 years, and a total of 14 peaks were identified in the FarmCPU model. Of them, two peaks, Chr8:4311475 (*p* = 1.50 × 10^–12^) and Chr6:2971050 (*p* = 6.74 × 10^–16^), were detected in both years, and were co-localized with the known Hd gene; the lead SNP Chr8:4311475 was located 22.3 kb away from the *Hd5* gene. When comparing the significant and major peaks identified by the two different models, only three known genes, *Hd3a*, *Hd5,* and *GF14-c*, were repeatedly detected as major candidate genes for Hd in both the MLM and FarmCPU models.

### Haplotype analysis of the three known Hd genes detected by GWAS

**FIGURE 3:**
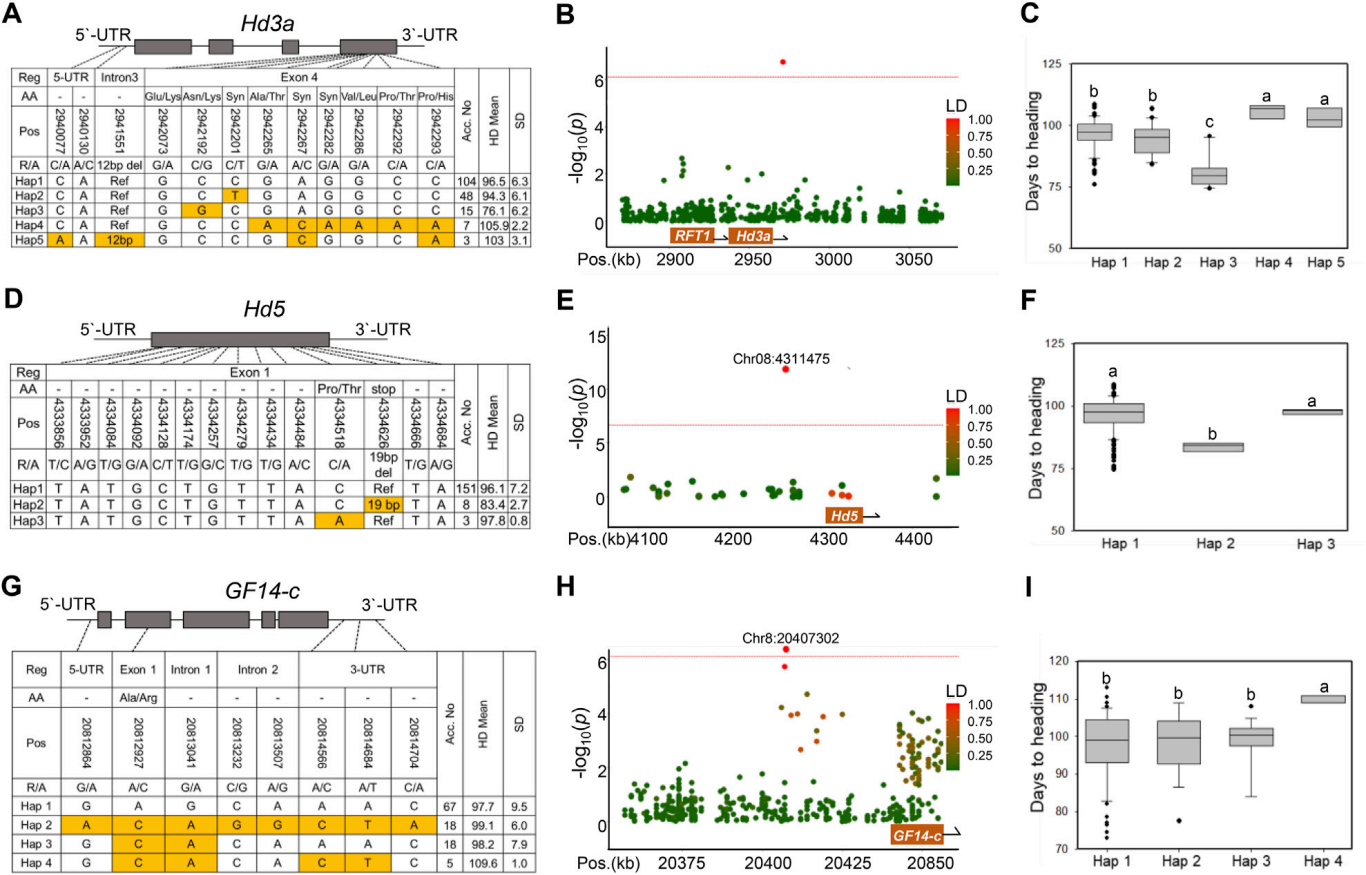
Haplotype analysis of the three known Hd genes (*Hd3a*, *Hd5,* and *GF14-c*) detected by GWAS. **(A)** Gene structure and haplotype analysis of *Hd3a* based on 11 significant SNPs and one InDel. A schematic diagram of *Hd3a* is shown on the top. **(B)** The LD plot shows association loci for *Hd3a* on chromosome 6. The color of each SNP indicates the *r* ^2^ value for the correlation with the lead SNP. Red and green color intensities indicate stronger and weaker LD (0–1). **(C)** Phenotypic variation of the five haplotypes for *Hd3a*. **(D)** Three haplotypes were detected in *Hd5* in CDS regions. **(E)** The LD plot shows association loci for *Hd5* on chromosome 8. **(F)** Phenotypic variation of the three haplotypes for *Hd5.* **(G)** Haplotype analysis of the peak associated with the *GF14-c* gene. **(H)** The LD plot shows association loci for *GF14-c* on chromosome 8. **(I)** Phenotypic variation of the four haplotypes for *GF14-c.* Different letters indicate significant differences according to one-way ANOVA and Duncan’s least significant range test (*p* < 0.05).

Haplotype and LD analyses identified known genes associated with rice Hd, such as *Hd3a*/*RFT1, Hd5,* and *GF14-c*. The haplotypes of the candidate genes were classified using non-synonymous SNPs and InDels in the coding sequence (CDS). *Hd3a* (LOC_Os06g06320), a major Hd gene in rice, was located approximately 28 kb away from the lead SNP Chr6:2971050 (*p* = 1.87 × 10^–7^) on chromosome 6 (Figure 3B). The genetic location of *Hd3a* included eight SNPs in the CDS, two SNPs in the 5′UTR, and one InDel in the intron region (Figure 3A). *Hd3a* was classified into five haplotypes. When comparing the mean Hd among haplotypes, the Hd of Hap three was significantly earlier than that of the other haplotypes. Haps one and two also showed intermediate Hd values, which were significantly earlier than those of Haps four and 5 (Figure 3C). Interestingly, we found an novel allele (Chr6:2942192) in Hap 3, which led to the amino acid substitution N145K (Asn/Lys) in the fourth exon of *Hd3a*. Fifteen rice accessions assigned to the Hap three group showed an extremely early heading phenotype (74–80 days) (Figure 3A). *Hd3a* promoter is a potential factor for generating diversity in flowering time (Takahashi et al., 2009). To test whether the Hap three allele is linked to the promoter allele variants, we analyzed approximately 2 kb of the promoter regions of *Hd3a* and identified three haplotypes. However, there was no significant association between the promoter sequence variants and the Hd phenotype (Supplementary Figure S3). We further investigated *Hd3a* expression levels in the leaves of heading plants. Hap three had a significantly higher *Hd3a* level than the other haplotypes (Supplementary Figure S4A). The primers used are listed in Supplementary Table S4. This result suggests that the Hap three allele may promote accelerated flowering by increasing *Hd3a* expression *via* an undetermined mechanism.

The second known Hd gene, *Hd5* (LOC_Os08g07740), was located about 22.3 kb away from the lead SNP Chr8:4311475 on chromosome 8 (Figure 3E). *Hd5*, consisting of only one exon, had 13 SNPs and one InDel in the coding region (Figure 3D). By haplotype analysis, *Hd5* was classified into three haplotype groups. The eight NK varieties belonging to Hap two showed a significant early heading phenotype compared with the other two haplotype groups. Of these allele variants, a 19 bp deletion (chr8:4334626) resulted in a frameshift (FS), causing loss-of-function of the *Hd5* gene (Figure 3F).

Another significant peak associated with Hd was detected on chromosome 8 (chr8:20408619) (Figures 2, 3H). *GF14-c*, acting as a negative regulator of flowering in rice (Purwestri et al., 2009), is located approximately 400 kb away from the lead SNP. The *GF14-c* (LOC_Os08g33370) haplotypes were constructed based on one SNP in a 5′UTR, one SNP in exon 1, three SNPs in the intron region, and three SNPs in the 3′UTR (Figure 3G). Four rice accessions were assigned to the Hap four group, which showed significantly delayed Hd (Figure 3I).

### Haplotype analyses of novel candidate genes for Hd phenotype

**FIGURE 4:**
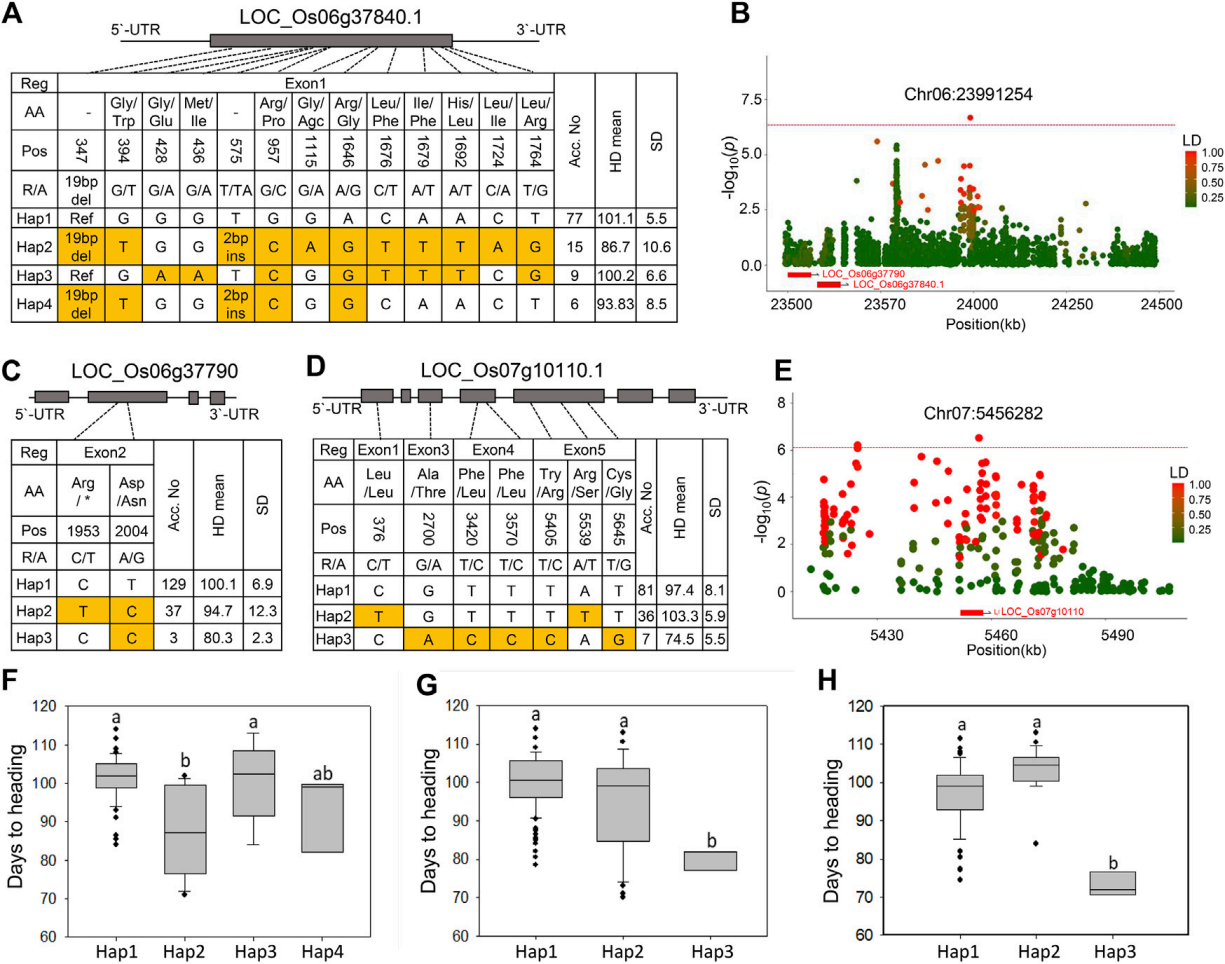
Haplotype analysis and strong association signals in chromosomes six and seven for Hd. **(A)** Four haplotypes were detected in LOC_Os06g37840. **(B)** The LD plot shows association loci for LOC_Os06g37840 and LOC_Os06g37790 at chromosome 6. The color of each SNP indicates *r* ^2^ (LD) for the correlation with the lead SNP. Red and green color intensities indicate stronger and weaker LD (0–1). **(C)** Two haplotypes were detected in LOC_Os06g37790. **(D)** Haplotype analysis and phenotypic differences between the HAPs of LOC_Os07g10110. **(E)** The LD plot shows association loci for LOC_Os07g10110 at chromosome 7. **(F–H)** Haplotype analysis of the LOC_Os06g37840 **(F)**, LOC_Os06g37790 **(G)**, and LOC_Os07g10110 **(H)**. Different letters indicate significant differences, according to Duncan’s multiple range test (*p* < 0.05).

Next, we performed LD and haplotype analysis for the three significant peaks identified in the MLM model in which Hd genes were not observed within the LD block (Chr4:15932637, Chr4:29201109, and Chr6:23991254) (Figure 2A). Haplotype analysis revealed a significant difference only between the haplotype groups associated with peak Chr6:23991254. Two unknown genes were identified 480–491 kb away from the lead SNPs Chr6:23991254 (Figure 4B). Of the two associated genes, LOC_Os06g37840.1 (a gene encoding a resistance protein) contains 11 SNPs and two InDels, and it was divided into four haplotype groups (Figure 4A). Haps two and four harbored a 19 bp deletion and two bp insertion in the first exon, and only Hap two showed a significant association with the early Hd phenotype (Figure 4F). The other gene, LOC_Os06g37790 (a gene encoding an expressed protein), had two SNPs in the second exon, and it was classified into three haplotypes (Figure 4C). Of these, Hap three was significantly associated with early Hd (Figure 4G).

We analyzed another 15 major peaks, although they were not significantly associated with the Hd phenotype based on the threshold line in the MLM model (Figures 2A,B). However, some of them showed significance in at least one of the yearly trials analyzed by the FarmCPU model. LD and haplotype analyses revealed that none showed significant association with Hd except for one peak chr7:5456828 (*p* = 3.02 × 10^−7^) where the LOC_Os07g10110.1 (a gene encoding expressed protein) was located 34.7 kb from the lead SNP (Figure 4E). Three haplotype patterns of this gene were identified in the NK population (Figure 4D). Of these, Hap three varieties showed significantly early heading compared to the other haplotypes (Figure 4H). This regulation indicates that the gene encoding the expressed protein can be used as a candidate gene for Hd.

### GWAS and haplotyping analysis for the PS phenotype

**FIGURE 5:**
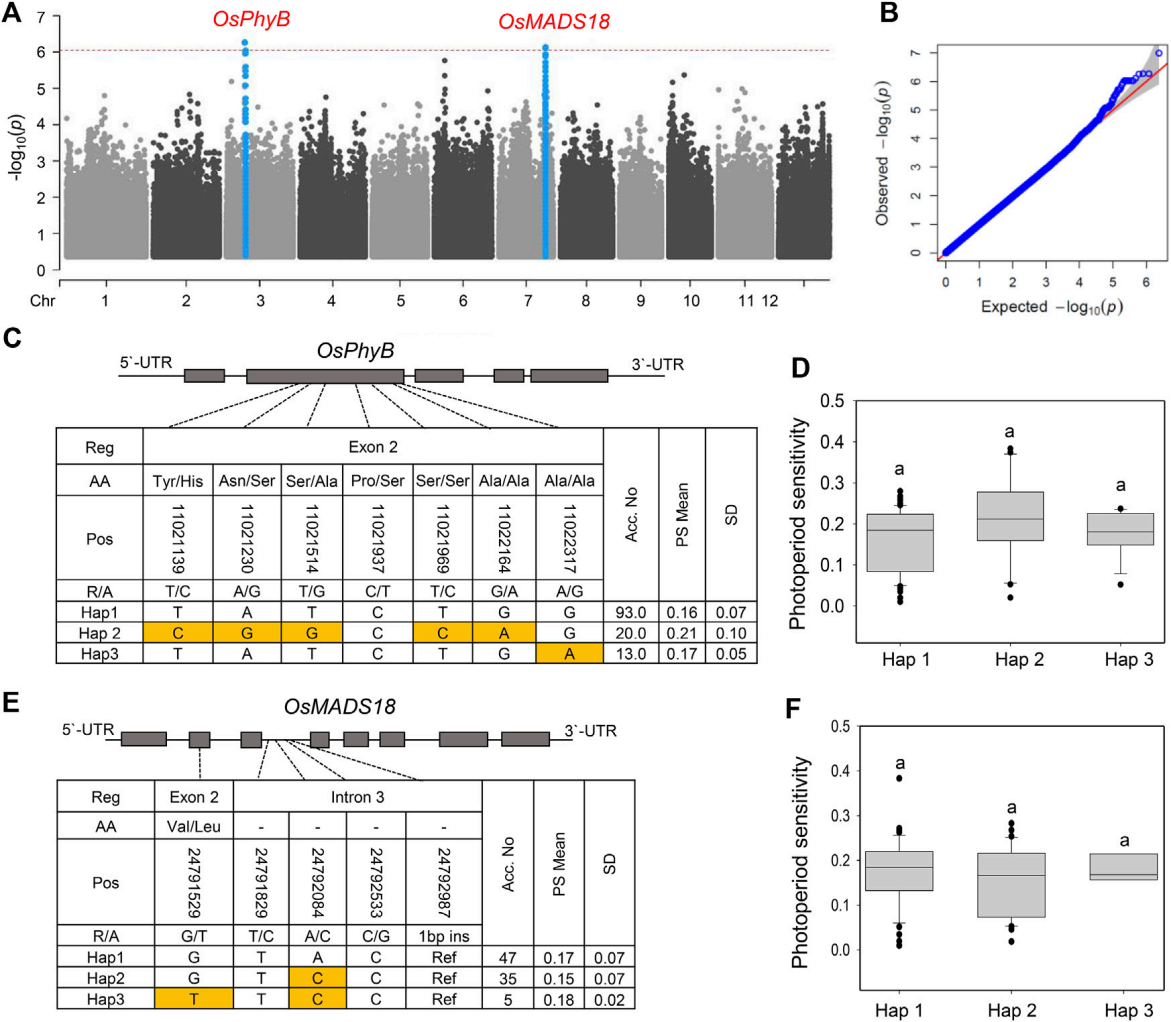
GWAS and haplotype analysis for photoperiod sensitivity. Manhattan. **(A)** and Q-Q plot **(B)** for a GWAS of photoperiod sensitivity in NK rice. For Q-Q plots, the horizontal and vertical axes represent the expected -log_10_ (*p*) and observed -log_10_ (*p*) of each SNP, respectively. The SNPs with *p*-values that deviated from the linear indicate reasonable positives. **(C)** *OsPhyB* haplotypes in CDS regions with SNPs. **(D)** Phenotypic variation of *OsPhyB* haplotypes. **(E)** Gene structure and haplotype analysis of *OsMADS18*. **(F)** Phenotypic variation of *OsMADS18* haplotypes. Different letters indicate significant differences according to Duncan’s multiple range test (*p* < 0.05).

Since the selected NK population was mainly photoperiod-insensitive, we next performed a GWAS for the PS phenotype. The photoperiod sensitivity of the NK population was evaluated as the difference in Hd between natural LD (Korea) and natural SD (Philippines). No significant peak was observed in the GWAS analysis for the PS phenotype; however, two major peaks were identified at chromosomes 3 and 7, although they were not over the threshold line in MLM (Figure 5A). Two SNPs, chr3:10130737 (*p* = 5.45 × 10^–7^) and chr7:25296269 (*p* = 7.87 × 10^–6^), were located 504–890 kb away from the *OsPhyB* and *OsMADS18* genes, respectively. *OsPhyB* delays heading in response to LD conditions (Lee et al., 2016), whereas *OsMADS18* promotes early heading (Wang et al., 2013). Haplotype analysis revealed that *OsPhyB* and *OsMADS18* could be classified into three main haplotypes (Figures 5C,E). There was no significant difference between haplotype groups for *OsPhyB* and *OsMADS18* (Figures 5D,F). These results indicate that known major PS genes were not detected in the GWAS analysis, possibly because the selected NK accessions were largely insensitive to photoperiod.

### Allelic combination of the major Hd genes contributed to PIS of NK varieties

**FIGURE 6:**
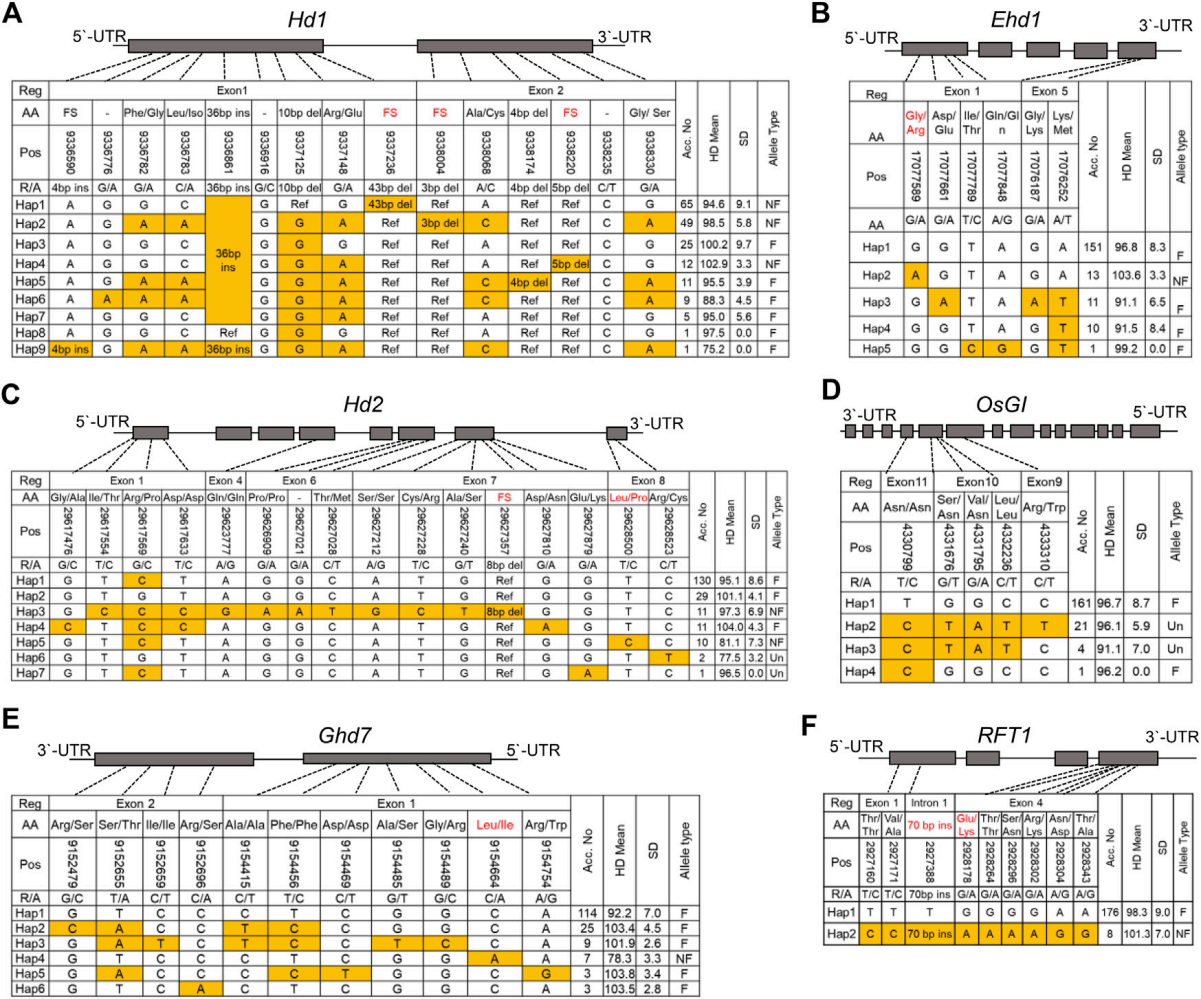
Haplotype analyses of the major Hd genes (*Hd1* **(A)**, *Ehd1* **(B)**, *Hd2* **(C)**, *OsGI* **(D)**, *Ghd7* **(E)** and *RFT1* **(F)**) with allelic variance (SNPs and InDels) in the CDS regions. The allelic frequencies in each rice type are presented in the columns towards the rights of the tables. Reference, Nipponbare (MSU).

To further elucidate the genetic composition underlying PIS in the NK population, we examined the haplotypes of additional major Hd genes that were not identified as significant loci for the Hd phenotype by GWAS in this study. Thus, we first performed haplotyping using allelic variations in the coding regions of *Hd1*, *OsGI*, *Ehd1*, *Hd2*, *Ghd7,* and *RFT1* (Figure 6). The allele types of each gene were divided into two groups, functional and nonfunctional alleles, based on their previously known protein functionality.

*Hd1*, one of the major PS-related genes, promotes flowering in SD, whereas it represses flowering under natural LD conditions (Yang et al., 2015). We found seven InDels and eight SNPs in the coding region of *Hd1*. Haplotype analysis classified the polymorphic loci of *Hd1* into nine groups (Figure 6A). Interestingly, Haps 1, 2, and 5 with 126 assigned rice varieties were nonfunctional (Figure 6A). The previously reported *Hd1* nonfunctional (NF) alleles were 43, 3, and four bp deletions in the CCT domain (Yano et al., 2000), and all of these alleles were identified in the NK varieties. The Hd of Hap nine occurred significantly earlier than that of the other haplotypes; however, only one variety belonged to Hap nine in the NK population (Supplementary Figure S5A).

*Ehd1*, a floral promoter under both SD and LD conditions, acts as a signal integrator that controls *Hd3a* and *RFT1* (Shrestha et al., 2014). The haplotypes of *Ehd1* were divided into five groups (Figure 6B). Only one NF allele (Chr10:17077589) was detected in Hap 2, and 13 varieties belonged to the NK population. Hap two showed a significantly delayed heading phenotype compared with the other haplotype groups (Supplementary Figure S5B).

*Hd2* is a floral repressor gene located on chromosome 7 (Zhang et al., 2016). Using haplotype analysis, 15 SNPs and one deletion were identified and classified into seven haplotype groups. Of these, Hap three and Hap five had known NF alleles on exon seven or eight of *Hd2* (Figure 6C). However, Hap five and Hap six showed a significantly early Hd phenotype compared to the other haplotypes (Supplementary Figure S5D). Ten and two varieties belonged to Hap five and Hap. 6, respectively. Based on this phenotype, it was inferred that Hap six might be an unknown NF allele of *Hd2*.

*OsGI* is a positive floral regulator of *Hd1* expression under SD and LD conditions (Hayama et al., 2003). Using haplotype analysis, five SNPs were identified in the CDS region of the *OsGI* gene and classified into four haplotype groups. The sequence profiles of Hap two and Hap three showed non-synonymous amino acid changes (Figure 6D); however, there was no significant difference between haplotype groups for the Hd phenotype (Supplementary Figure S5C).

*Ghd7* contained 11 SNPs and was classified into six haplotypes. Among the haplotypes, Hap three contained one NF allele that showed significantly earlier than that of the other haplotypes. (Supplementary Figure S5F). Among the rice accessions of NK, seven varieties had a *Ghd7-0* allele caused by an SNP (Chr7:9154664), and the Hd was significantly earlier, by 22°days on average, than that of the varieties with a functional allele (Figure 6E).

Haplotype analysis of *RFT1,* a flowering promoter, identified nine polymorphic loci and classified these allelic variants into two haplotypes. Hap two harbored two NF alleles, a 70 bp insertion in an intron and SNP chr6:2928178, causing amino acid substitution Gly/Lys (E105K); however, no significant difference in Hd was observed between haplotypes (Supplementary Figure S5E).

Haplotype analysis of the major Hd genes for the Hd phenotype revealed that four Hd genes (*Hd1*, *Ehd1*, *Hd2*, and *Ghd7*) unexpectedly showed significant differences between haplotypes. These results indicate that some of the major Hd genes still regulate the Hd phenotype in this population; however, the allele effect of these Hd genes was not observed in GWAS because these were mostly rare alleles that were filtered out by the MAF parameters (≥0.05).

**FIGURE 7:**
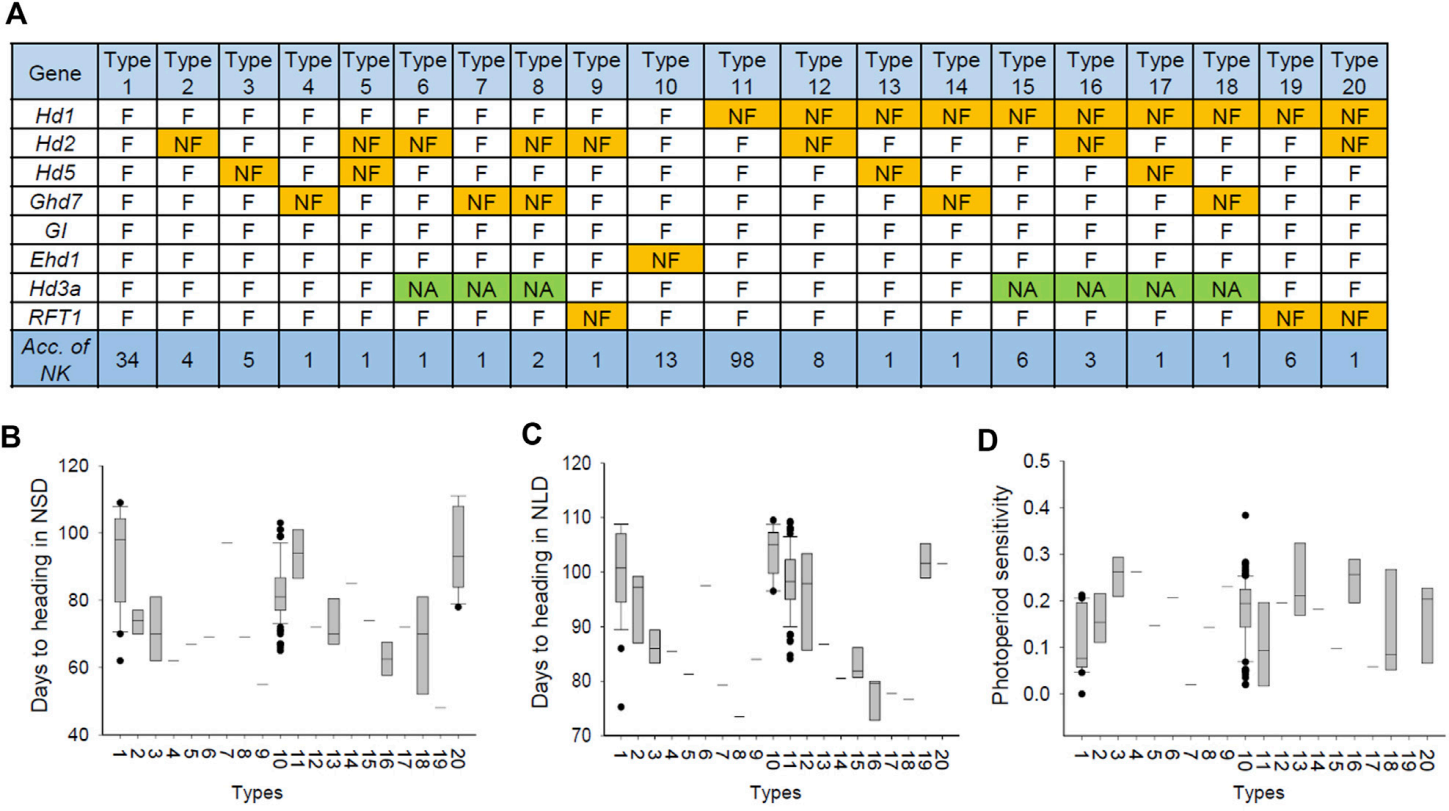
Allelic composition of major Hd genes regulating days to heading in the 190 NK rice accessions. **(A)** Haplotype combination of major Hd genes. **(B)** Phenotypic variation between the types of Hd genes combination in NSD conditions. **(C)** Phenotypic variation between the types of Hd genes combination in NLD conditions. **(D)** Phenotypic variation between the types of Hd genes combination in PS.

The Hd pathway is a complex mechanism that is explained by intergenic interactions. Thus, we further analyzed the allele combinations of eight major Hd genes to reveal the combined effect of Hd genes on PIS in rice. The allele of each Hd gene was functional and nonfunctional, and these allele combinations were classified into 20 different types (Figure 7A). The Hd patterns of each type were similar under the natural SD and LD conditions (Figures 7B,C). The PS value analysis revealed that all 20 types showed a low PS index value (PS < 0.3) in the NK population, indicating that all types were largely photoperiod-insensitive (Figure 7D).

The number of varieties carrying NF alleles was the largest for *Hd1* (126 varieties), followed by *Hd2* (21), *Ehd1* (13), *Hd5* (8), *RFT1* (8), and *Ghd7* (7) (Figure 7A). Of the 64 varieties that did not carry the *Hd1* nonfunctional allele, 30 varieties harbored at least one of the NF alleles of the other major Hd genes. The other 34 varieties carried only functional alleles for eight major Hd genes. This result indicated that *Hd1* mainly contributed, and other Hd genes, such as *Hd2* and *Ehd1*, partially contributed to PIS in the NK population.

Type 1, carrying only functional alleles for the Hd genes, showed the second-most delayed Hd phenotype, with only type 10 showing an even later heading. This may be due to the NF allele of *Ehd1*, a floral promoter. Type 11, to which 98 cultivars (57% of the total) belonged, had a NF allele of *Hd1* and showed late heading. It seems that the NF alleles of *Hd2* (types 2, 5, 6, 8, 9, 12, 16, and 20), *Hd5* (types 3, 5, 13, and 17), and *Ghd7* (types 4, 7, 8, 14, and 18), which are floral inhibitory genes, mostly contributed to promoting heading compared to types 1 and 11. Notably, a new allele of *Hd3a* was observed in types 6–8 and 15–18, showing a remarkably early heading and suggesting that this is attributed to the novel allele of *Hd3a*. Type 8, which had a combination of NF alleles of *Ghd7* and *Hd2* and novel alleles of *Hd3a*, showed the earliest heading (70–72°days). Taken together, these results suggest that the allelic combination of major Hd genes, including floral inhibitory and promoting genes, contributed to days to heading, and PIS was mainly attributed to the NF alleles in the NK population.

### Geographical distribution of the varieties with 20 different allelic combination types of the major Hd genes

**FIGURE 8:**
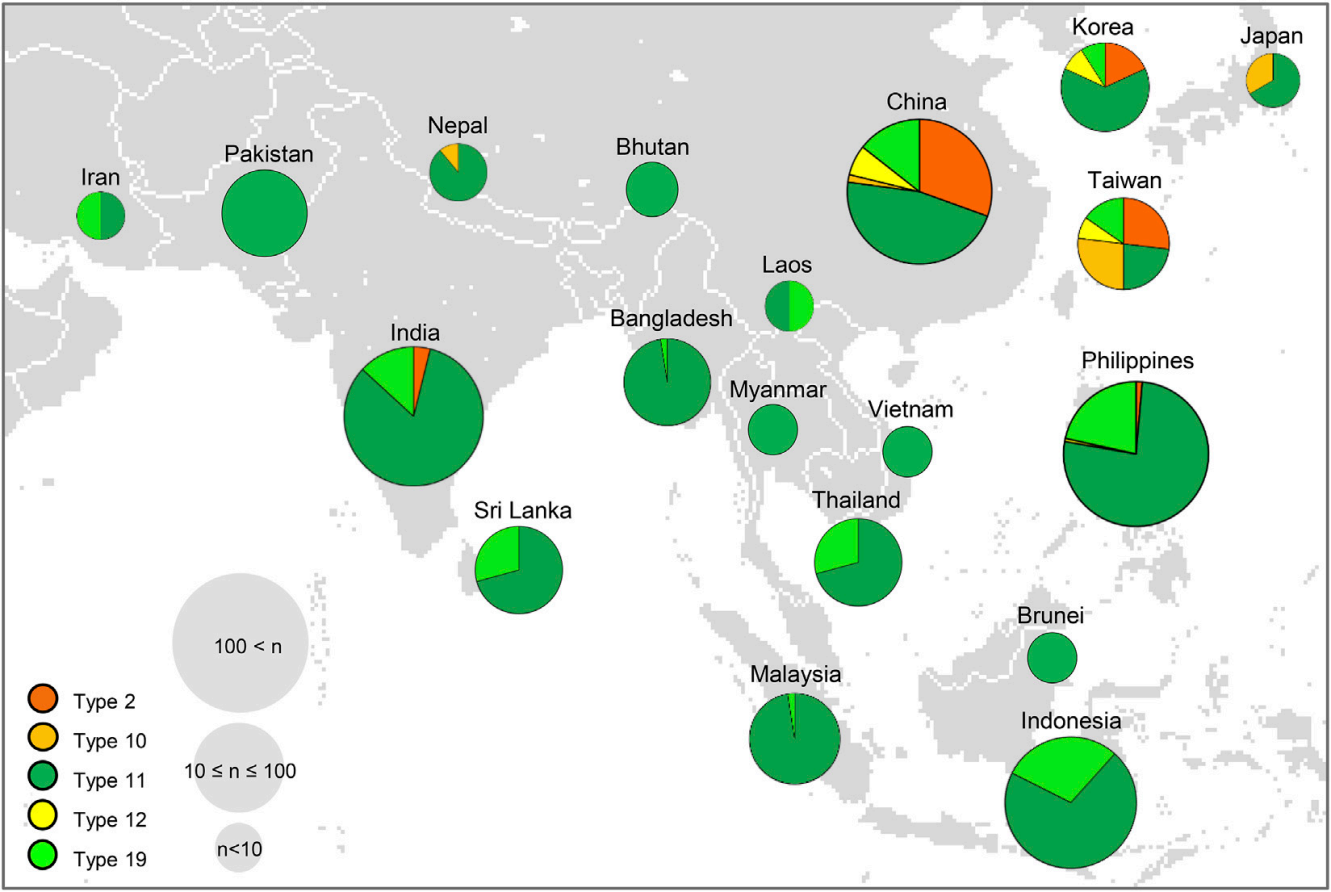
Geographic distribution of cultivated rice varieties harboring different allelic combination types of major Hd genes in Asia. Each type is shown as different colors in the pie chart; Type 2 (orange), Type 10 (mustard), Type 11 (green), Type 12 (yellow), Type 19 (light green). The size of the circles represents the number of varieties in each country. In total, 3,000 rice varieties in the International Rice Genebank Collection Information System were used for allelic analysis.

We further investigated the geographical distribution of the 20 different allele combinations using 3 K-RGP (Mansueto et al., 2017). Among the 20 types, we mapped the geographical distribution of haplotypes 2, 10, 11, 12, and 19, which were found in 3 K-RGP, except for type 1, which harbored only functional alleles of the Hd genes (Figure 7A). Type 2 (harboring *Hd2* NF allele) was found in 54 rice accessions in the 3 K-RGP and was mainly distributed in temperate regions (latitude, 270–55°), including China, Korea, and Nepal. Although not mapped in Figure 8 (Asian map), type 4 (harboring *Ghd7* NF allele) and type 6 (harboring NF alleles of *Hd2* and the novel allele of *Hd3a*) were mainly distributed in high latitude regions such as New Zealand, Bulgaria, and Hungary (latitude, 350–48°). These results indicate that varieties with NF alleles of floral repressor genes, such as *Hd2* and *Ghd7,* were largely distributed in high-latitude regions. In contrast, the varieties belonging to type 11 with the *Hd1* NF allele were found in 570 accessions of the 3 K-RGP and were widely distributed throughout Asia. Interestingly, type 12, which has both *Hd1* and *Hd2* NF alleles, is distributed at relatively high latitudes in mainland China, Taiwan, and Korea. This suggests that the *Hd2* NF allele contributes to rice cultivation at high latitudes, irrespective of the presence of *Hd1* allele. Therefore, the geographic distribution of a combination of major Hd gene haplotypes revealed that *Hd1* NF allele contributed to broad area rice cultivation ranging from low to high latitudes. In contrast, NF alleles of floral repressors such as *Hd2* and *Ghd7* enabled rice cultivation at high latitudes.

## Discussion

We performed GWAS analysis using MLM and FarmCPU models for the Hd and PS phenotypes to elucidate the genetic composition of photoperiod-insensitive NK rice. As expected, most of the major Hd genes were not significantly identified as associated signals in the GWAS. However, several SNPs significantly associated with Hd traits were still identified near the Hd gene regions, such as Chr6:2971050 and *Hd3a*. Five significant loci linked to known Hd (*Hd3a, Hd5,* and *Gf14c*) or novel genes were identified for heading date. Haplotyping of candidate genes located within the LD of the major peaks, including non-significant peaks, revealed that three known Hd genes and three novel genes (derived from two major peaks) were significantly associated with the Hd phenotype. Through further GWAS analysis of the PS phenotype, we also found *OsPhyB* and *OsMADS18* genes linked to two major peaks, but their haplotypes were not significantly associated with the PS trait. This may be attributed to the population stratification of NK varieties previously selected for normal heading dates and growth in natural SD conditions (Philippines).

*Hd3a* and *Hd5* played an important role in Hd regulation in the selected NK rice population (Figures 2A–F). Using GWAS, *Hd3a* was detected as a major genetic factor associated with the Hd phenotype in both trials with the NK population. *Hd3a* was located 28 kb away from lead SNP Chr6:2971050 (Figures 2, ). The lead SNP in the locus correlated well with the SNP Chr6:2942192, located in exon four of *Hd3a*, which was previously presented as a novel allele (Li et al., 2020). The 15 varieties belonging to Hap three harboring this allele showed significantly earlier Hd than the other haplotypes, including the functional allele (Figures 3A,D), suggesting that this allele is hyperfunctional.

Since *Hd3a* expression levels are strongly correlated with flowering time (Takahashi et al., 2009), we compared mRNA expression levels of the *Hd3a* haplotypes. Hap three was significantly higher in *Hd3a* level compared to the other haplotypes (Supplemental figure S4A). Further comparison of *Hd3a* expression in the selected varieties of Hap one and Hap 3 with the same heading date (78 days to heading) revealed that Hap three varieties still showed significantly earlier heading than Hap 1 (harboring a functional allele) (Supplementary Figure S4B). This suggests that Hap three is the hyper functional haplotype of *Hd3a*. Since a promoter variation of the *Hd3a* gene was not significantly correlated with the Hd phenotype (Supplementary Figure S3), it is inferred that SNP (Chr6:2942192) resulting in the amino acid substitution N145K (Asp/Lys) allele could contribute to the upregulation of *Hd3a* expression through mechanisms that has not yet been elucidated, other than promoter variation. This merits a further functional study of this hyper-functional allele, SNP (Chr6:2942192), in the fourth exon of the *Hd3a* gene.

Another major gene, *Hd5*, a loss-of-function allele caused by a 19 bp deletion (chr8:4334626), was found in eight out of 190 NK varieties (Figure 4A). This NF allele causing early flowering has been extensively identified in rice varieties growing in Hokkaido, a northern region of Japan (Fujino et al., 2013). *Hd5* acts as a floral repressor under LD conditions and delays flowering by downregulating the expression of *Ehd1*, *Hd3a*, and *RFT1* (Wei et al., 2010). The NF alleles of *Hd5* and hyper-functional alleles of *Hd3a* were found in 21 varieties (11% in frequency) in the NK population, causing an early heading date. PS and the transition from the vegetative to reproductive phase can be explained by different allele combinations of the major Hd genes in rice accessions. Several major genes that control Hd in response to photoperiod have been identified and described (Yamamoto et al., 1998). Three of them (*Hd1*, *Hd2*, and *Hd5*) have been shown to suppress flowering under LD conditions (Yano et al., 2000; Kojima et al., 2002; Xue et al., 2008; Wei et al., 2010; Yan et al., 2011; Fujino et al., 2013). It has been reported that PIS is generated by loss-of-function or weak alleles of Hd genes, enabling their distribution to specific environmental conditions, such as high latitudes (Gouesnard et al., 2002; Turner et al., 2005; Murphy et al., 2011; Goretti et al., 2017). NK varieties carried nonfunctional *Hd1*, *Hd2*, *Ghd7,* and *Hd5* alleles, and those with a small PS index (<0.3) were less photoperiod-sensitive (Figures 7A,D). A photoperiod-sensitivity index close to zero is considered photoperiod-insensitive (Poonyarit, 1987).

Rice varieties carrying NF alleles of *Hd1* can be cultivated in a wide range of regions because of the derepression of *Hd3a.* This allows flowering under a non-inductive photoperiod (Takahashi et al., 2009; Wu et al., 2020). The nonfunctional allele of *Hd1* plays a major role in adaptation to various areas of rice cultivation (Fujino et al., 2013). The most frequent NF allele type in *Hd1* was the 43 bp deletion (se1 type) (Yano et al., 2000), which was found in 65 NK varieties in this study. The second one was a four bp deletion in the exon (Takahashi et al., 2009), carried by 49 NK varieties (Figure 6A). A total of 126 varieties carried NF alleles of *Hd1*, indicating that 66% of the NK varieties were affected by the *Hd1* allele and were less sensitive to photoperiods. This supports the hypothesis that the *Hd1* nonfunctional allele plays an important role in the regional adaptation of rice.

The other major Hd genes, including *Ehd1, Hd2, Ghd7, Hd5,* and *RFT1,* also partially contributed to lower PS in the NK varieties*.* A total of 156 NK varieties carried at least one NF allele of these Hd genes. It is possible that the other minor Hd genes, which were not analyzed in our study, contributed to the lower PS of the 34 varieties carrying only functional alleles of major Hd genes.

A global map with the regional distributions of the five different allele combination types obtained from the 3 K-RGP showed that rice varieties with the *Hd1* NF allele were widely distributed from low to high latitudes (Figure 8). *Hd1* NF alleles may have occurred first in the insular region of Southeast Asia, introgressed to many local landraces, and moved to other regions (Wu et al., 2020). Kim et al. (2018) reported that NF alleles of *Hd1* might enable the breeding of temperate Japonica varieties adapted to tropical regions. The NF allele *Hd1* caused by the 43 bp deletion in exon 1, has been widely utilized in rice breeding in Japan, China, and Europe (Gómez-Ariza et al., 2015; Fujino et al., 2019; Leng et al., 2020). These results indicated that the NF alleles of *Hd1* play an important role in rice cultivation over a wide range of latitudes.

Meanwhile, NF alleles of floral repressors were mainly found in high-latitude regions when analyzed with rice information obtained from the 3 K-RGP (Figure 8). It was previously reported that varieties with NF alleles of floral repressing genes are locally distributed in the northern regions of rice cultivation, including China, the Korean Peninsula, Japan, and Europe. *Hd2* single or *Hd2*/*Ghd7* double mutations were found in japonica rice cultivated in high-latitude regions of northeastern Asia (Koo et al., 2013). The NF allele of *Ghd7* is important for extremely early heading in varieties from Hokkaido and Europe (Fujino and Sekiguchi, 2005; Nonoue et al., 2008; Xue et al., 2008; Shibaya et al., 2011). Furthermore, the NF allele of *Hd5* (19 bp deletion) contributed to the adaptation of rice cultivars to the northern limit of rice cultivation in Hokkaido, Japan (Fujino et al., 2013). These reports indicate that NF alleles of floral repressors play an important role in rice adaptation to high latitudes. In conclusion, the allele combination of *Hd1* and the other floral repressing Hd genes could contribute to the specific adaptation of rice cultivation to low-to higher-latitude regions with no limitation of photoperiod. The PIS of the NK rice population used in this study was mainly attributed to the NF allele of *Hd1* and partially to the alleles of other floral repressors (such as *Hd2*, *Hd5*, and *Ghd7*) and floral accelerators (such as *Hd3a*, *Ehd1* and *RFT1*). Further analysis of 34 varieties belonging to type 1 harboring only functional alleles of the Hd genes investigated would help reveal the role of the other Hd genes in the PIS of the NK varieties. The PIS NK varieties and their genetic information can be used as valuable materials for local adaptation to rice breeding in the future.

## Data Availability

The data presented in the study are deposited in the Sequence Read Archive (SRA) of the NCBI repository, accession number PRJNA896894.

## Glossary

GWAS: Genome-wide association study
PS: Photoperiod sensitivity
PIS: Photoperiod insensitivity
Hap: Haplotype
LD: Long day
SD: Short day
SNP: Single nucleotide polymorphism
InDel: Insertion deletion
Hd: Heading date
NK: North Korea
DPRK: Democratic People’s Republic of Korea
IRRI: International Rice Research Institute
MLM: Mixed linear models
rMVP: Memory-efficient, Visualization-enhanced, and Parallel-accelerated R package
FarmCPU: Fixed and random model Circulating Probability Unification
LD: Linkage disequilibrium
MAF: Minor allele frequency
PCA: Principal component analysis
CDS: Coding sequence
UTR: Untranslated region
*Hd1*: Heading date one
*Hd2*: Heading date two
*Hd3a*: Heading date 3a
*Hd5*: Heading date five
*Ghd7*: Heading date seven
*Ehd1*: Early heading one
*RFT1*: Rice flowering locus T
*GI*: GIGANTEA
*OsPhyB*: Phytochrome B
QTL: Quantitative trait locus
Chr: Chromosome
Pos: Position
NJ tree: Neighbor-joining tree
VCF: Variant call format
MSU: Michigan State University
FS: Frameshift
3K-RGP: 3000 Rice Genome Project database
NF: Nonfunctional

## References

[B1] Corbesier L. Vincent C. Jang S. Fornara F. Fan Q. Searle I. (2007). FT protein movement contributes to long-distance signaling in floral induction of Arabidopsis. Science 316 (5827), 1030–1033. 10.1126/science.1141752 17446353

[B2] Cubry P. Pidon H. Ta K. N. Tranchant-Dubreuil C. Thuillet A. C. Holzinger M. (2020). Genome wide association study pinpoints key agronomic QTLs in african rice Oryza glaberrima. Rice 13 (1), 66. 10.1186/s12284-020-00424-1 32936396 PMC7494698

[B3] Doi K. Izawa T. Fuse T. Yamanouchi U. Kubo T. Shimatani Z. (2004). Ehd1, a B-type response regulator in rice, confers short-day promotion of flowering and controls FT-like gene expression independently of Hd1. Genes. Dev. 18 (8), 926–936. 10.1101/gad.1189604 15078816 PMC395851

[B4] Famoso A. N. Zhao K. Clark R. T. Tung C. W. Wright M. H. Bustamante C. (2011). Genetic architecture of aluminum tolerance in rice (Oryza sativa) determined through genome-wide association analysis and QTL mapping. PLoS Genet. 7 (8), e1002221. 10.1371/journal.pgen.1002221 21829395 PMC3150440

[B5] Fujino K. Obara M. Ikegaya T. (2019). Establishment of adaptability to the northern-limit of rice production. Mol. Genet. Genomics 294 (3), 729–737. 10.1007/s00438-019-01542-2 30874890

[B6] Fujino K. (2003). Photoperiod sensitivity gene controlling heading date in rice cultivars in the northernmost region of Japan. Euphytica 131 (1), 97–103. 10.1023/A:1023088810701

[B7] Fujino K. Sekiguchi H. (2005). Mapping of QTLs conferring extremely early heading in rice (Oryza sativa L.) Theor. Appl. Genet. 111 (2), 393–398. 10.1007/s00122-005-2035-3 15940510

[B8] Fujino K. Yamanouchi U. Yano M. (2013). Roles of the Hd5 gene controlling heading date for adaptation to the northern limits of rice cultivation. Theor. Appl. Genet. 126 (3), 611–618. 10.1007/s00122-012-2005-5 23090144

[B9] Gómez-Ariza J. Galbiati F. Goretti D. Brambilla V. Shrestha R. Pappolla A. (2015). Loss of floral repressor function adapts rice to higher latitudes in Europe. J. Exp. Bot. 66 (7), 2027–2039. 10.1093/jxb/erv004 25732533 PMC4378634

[B10] Goretti D. Martignago D. Landini M. Brambilla V. Gómez-Ariza J. Gnesutta N. (2017). Transcriptional and post-transcriptional mechanisms limit heading date 1 (Hd1) function to adapt rice to high latitudes. PLoS Genet. 13 (1), e1006530. 10.1371/journal.pgen.1006530 28068345 PMC5221825

[B11] Gouesnard B. Rebourg C. Welcker C. Charcosset A. (2002). Analysis of photoperiod sensitivity within a collection of tropical maize populations. Genet. Resour. Crop Evol. 49 (5), 471–481. 10.1023/A:1020982827604

[B12] Hayama R. Yokoi S. Tamaki S. Yano M. Shimamoto K. (2003). Adaptation of photoperiodic control pathways produces short-day flowering in rice. Nature 422 (6933), 719–722. 10.1038/nature01549 12700762

[B13] Huang X. Zhao Y. Wei X. Li C. Wang A. Zhao Q. (2011). Genome-wide association study of flowering time and grain yield traits in a worldwide collection of rice germplasm. Nat. Genet. 44 (1), 32–39. 10.1038/ng.1018 22138690

[B14] Izawa T. (2007). Adaptation of flowering-time by natural and artificial selection in Arabidopsis and rice. J. Exp. Bot. 58 (12), 3091–3097. 10.1093/jxb/erm159 17693414

[B15] Jaeger K. E. Wigge P. A. (2007). FT protein acts as a long-range signal in arabidopsis. Curr. Biol. 17 (12), 1050–1054. 10.1016/j.cub.2007.05.008 17540569

[B16] Kim S. K. Park H. Y. Jang Y. H. Lee K. C. Chung Y. S. Lee J. H. (2016). OsNF-YC2 and OsNF-YC4 proteins inhibit flowering under long-day conditions in rice. Planta 243 (3), 563–576. 10.1007/s00425-015-2426-x 26542958

[B17] Kim S. R. Torollo G. Yoon M. R. Kwak J. Lee C. K. Prahalada G. D. (2018). Loss-of-Function alleles of heading date 1 (Hd1) are associated with adaptation of temperate japonica rice plants to the tropical region. Front. Plant Sci. 9, 1827. 10.3389/fpls.2018.01827 30619400 PMC6295564

[B18] Kojima S. Takahashi Y. Kobayashi Y. Monna L. Sasaki T. Araki T. (2002). Hd3a, a rice ortholog of the Arabidopsis FT gene, promotes transition to flowering downstream of Hd1 under short-day conditions. Plant Cell. Physiol. 43 (10), 1096–1105. 10.1093/pcp/pcf156 12407188

[B19] Komiya R. Ikegami A. Tamaki S. Yokoi S. Shimamoto K. (2008). Hd3a and RFT1 are essential for flowering in rice. Development 135 (4), 767–774. 10.1242/dev.008631 18223202

[B20] Koo B. H. Yoo S. C. Park J. W. Kwon C. T. Lee B. D. An G. (2013). Natural variation in OsPRR37 regulates heading date and contributes to rice cultivation at a wide range of latitudes. Mol. Plant 6 (6), 1877–1888. 10.1093/mp/sst088 23713079

[B21] Kumar S. Stecher G. Li M. Knyaz C. Tamura K. (2018). Mega X: Molecular evolutionary genetics analysis across computing platforms. Mol. Biol. Evol. 35 (6), 1547–1549. 10.1093/molbev/msy096 29722887 PMC5967553

[B22] Kwon C. T. Yoo S. C. Koo B. H. Cho S. H. Park J. W. Zhang Z. (2014). Natural variation in Early flowering1 contributes to early flowering in japonica rice under long days. Plant Cell. Environ. 37 (1), 101–112. 10.1111/pce.12134 23668360

[B23] Lee Y. S. Yi J. An G. (2016). OsPhyA modulates rice flowering time mainly through OsGI under short days and Ghd7 under long days in the absence of phytochrome B. Plant Mol. Biol. 91 (4-5), 413–427. 10.1007/s11103-016-0474-7 27039184

[B24] Leng Y. Gao Y. Chen L. Yang Y. Huang L. Dai L. (2020). Using Heading date 1 preponderant alleles from indica cultivars to breed high-yield, high-quality japonica rice varieties for cultivation in south China. Plant Biotechnol. J. 18 (1), 119–128. 10.1111/pbi.13177 31141272 PMC6920332

[B25] Li J. Y. Wang J. Zeigler R. S. (2014). The 3, 000 rice genomes project: New opportunities and challenges for future rice research. Gigascience 3, 8. 10.1186/2047-217x-3-8 24872878 PMC4035671

[B26] Li X. Chen Z. Zhang G. Lu H. Qin P. Qi M. (2020). Analysis of genetic architecture and favorable allele usage of agronomic traits in a large collection of Chinese rice accessions. Sci. China. Life Sci. 63 (11), 1688–1702. 10.1007/s11427-019-1682-6 32303966

[B27] Lin H. X. Yamamoto T. Sasaki T. Yano M. (2000). Characterization and detection of epistatic interactions of 3 QTLs, Hd1, Hd2, and Hd3, controlling heading date in rice using nearly isogenic lines. Theor. Appl. Genet. 101 (7), 1021–1028. 10.1007/s001220051576

[B28] Lipka A. E. Tian F. Wang Q. Peiffer J. Li M. Bradbury P. J. (2012). Gapit: Genome association and prediction integrated tool. Bioinformatics 28 (18), 2397–2399. 10.1093/bioinformatics/bts444 22796960

[B29] Liu C. Tu Y. Liao S. Fu X. Lian X. He Y. (2021). Genome-wide association study of flowering time reveals complex genetic heterogeneity and epistatic interactions in rice. Gene 770, 145353. 10.1016/j.gene.2020.145353 33333227

[B30] Ma X. Feng F. Wei H. Mei H. Xu K. Chen S. (2016). Genome-wide association study for plant height and grain yield in rice under contrasting moisture regimes. Front. Plant Sci. 7, 1801. 10.3389/fpls.2016.01801 27965699 PMC5126757

[B31] Mansueto L. Fuentes R. R. Borja F. N. Detras J. Abriol-Santos J. M. Chebotarov D. (2017). Rice SNP-seek database update: New SNPs, indels, and queries. Nucleic Acids Res. 45 (D1), D1075–D1081. 10.1093/nar/gkw1135 27899667 PMC5210592

[B32] Mathieu J. Warthmann N. Küttner F. Schmid M. (2007). Export of FT protein from phloem companion cells is sufficient for floral induction in arabidopsis. Curr. Biol. 17 (12), 1055–1060. 10.1016/j.cub.2007.05.009 17540570

[B33] Murphy R. L. Klein R. R. Morishige D. T. Brady J. A. Rooney W. L. Miller F. R. (2011). Coincident light and clock regulation of pseudoresponse regulator protein 37 (PRR37) controls photoperiodic flowering in sorghum. Proc. Natl. Acad. Sci. U. S. A. 108(39), 16469–16474. 10.1073/pnas.1106212108 21930910 PMC3182727

[B34] Murray M. G. Thompson W. F. (1980). Rapid isolation of high molecular weight plant DNA. Nucleic Acids Res. 8 (19), 4321–4325. 10.1093/nar/8.19.4321 7433111 PMC324241

[B35] Nayyeripasand L. Nematzadeh G. Jelodar N. Ahmadikhah A. Azimi M. (2013). Development of an allele-specific functional marker for studying Hd1 effect on flowering time of rice. Int. J. basic Appl. Sci. 4, 2209–2215.

[B36] Nonoue Y. Fujino K. Hirayama Y. Yamanouchi U. Lin S. Y. Yano M. (2008). Detection of quantitative trait loci controlling extremely early heading in rice. Theor. Appl. Genet. 116 (5), 715–722. 10.1007/s00122-007-0704-0 18193402

[B37] Poonyarit ม. (1987). Photoperiod-sensitivity index of five parents and ten F1 hybrids of rice under natural daylength. Thai Agri. Res. J. 5 (1-3), 9–15.

[B38] Purcell S. Neale B. Todd-Brown K. Thomas L. Ferreira M. A. Bender D. (2007). Plink: A tool set for whole-genome association and population-based linkage analyses. Am. J. Hum. Genet. 81 (3), 559–575. 10.1086/519795 17701901 PMC1950838

[B39] Purwestri Y. A. Ogaki Y. Tamaki S. Tsuji H. Shimamoto K. (2009). The 14-3-3 protein GF14c acts as a negative regulator of flowering in rice by interacting with the florigen Hd3a. Plant Cell. Physiol. 50 (3), 429–438. 10.1093/pcp/pcp012 19179350

[B40] Shibaya T. Nonoue Y. Ono N. Yamanouchi U. Hori K. Yano M. (2011). Genetic interactions involved in the inhibition of heading by heading date QTL, Hd2 in rice under long-day conditions. Theor. Appl. Genet. 123 (7), 1133–1143. 10.1007/s00122-011-1654-0 21789706

[B41] Shrestha R. Gómez-Ariza J. Brambilla V. Fornara F. (2014). Molecular control of seasonal flowering in rice, Arabidopsis and temperate cereals. Ann. Bot. 114, 1445–1458. 10.1093/aob/mcu032 24651369 PMC4204779

[B42] Takahashi Y. Teshima K. M. Yokoi S. Innan H. Shimamoto K. (2009). Variations in Hd1 proteins, Hd3a promoters, and Ehd1 expression levels contribute to diversity of flowering time in cultivated rice. Proc. Natl. Acad. Sci. U. S. A. 106 (11), 4555–4560. 10.1073/pnas.0812092106 19246394 PMC2647979

[B43] Tamaki S. Matsuo S. Wong H. L. Yokoi S. Shimamoto K. (2007). Hd3a protein is a mobile flowering signal in rice. Science 316 (5827), 1033–1036. 10.1126/science.1141753 17446351

[B44] Tanaka N. Shenton M. Kawahara Y. Kumagai M. Sakai H. Kanamori H. (2020). Investigation of the genetic diversity of a rice core collection of Japanese landraces using whole-genome sequencing. Plant Cell. Physiol. 61 (12), 2087–2096. 10.1093/pcp/pcaa125 PMC7861467 33539537

[B45] Turner A. Beales J. Faure S. Dunford R. P. Laurie D. A. (2005). The pseudo-response regulator ppd-H1 provides adaptation to photoperiod in barley. Science, 310(5750), 1031–1034. 10.1126/science.1117619 16284181

[B46] Wang J.-D. Lo S.-F. Li Y.-S. Chen P.-J. Lin S.-Y. Ho T.-Y. (2013). Ectopic expression of OsMADS45 activates the upstream genes Hd3a and RFT1 at an early development stage causing early flowering in rice. Bot. Stud. 54 (1), 12. 10.1186/1999-3110-54-12 28510861 PMC5432754

[B47] Wang P. Gong R. Yang Y. Yu S. (2019). Ghd8 controls rice photoperiod sensitivity by forming a complex that interacts with Ghd7. BMC Plant Biol. 19 (1), 462. 10.1186/s12870-019-2053-y 31675987 PMC6825352

[B48] Wei X. Xu J. Guo H. Jiang L. Chen S. Yu C. (2010). DTH8 suppresses flowering in rice, influencing plant height and yield potential simultaneously. Plant Physiol. 153 (4), 1747–1758. 10.1104/pp.110.156943 20566706 PMC2923886

[B49] Wu C. C. Wei F. J. Chiou W. Y. Tsai Y. C. Wu H. P. Gotarkar D. (2020). Studies of rice Hd1 haplotypes worldwide reveal adaptation of flowering time to different environments. PLoS One 15 (9), e0239028. 10.1371/journal.pone.0239028 32941524 PMC7498076

[B50] Xue W. Xing Y. Weng X. Zhao Y. Tang W. Wang L. (2008). Natural variation in Ghd7 is an important regulator of heading date and yield potential in rice. Nat. Genet. 40 (6), 761–767. 10.1038/ng.143 18454147

[B51] Yamamoto T. Kuboki Y. Lin S. Y. Sasaki T. Yano M. (1998). Fine mapping of quantitative trait loci Hd-1, Hd-2 and Hd-3, controlling heading date of rice, as single Mendelian factors. Theor. Appl. Genet. 97 (1-2), 37–44. 10.1007/s001220050864

[B52] Yan W. H. Wang P. Chen H. X. Zhou H. J. Li Q. P. Wang C. R. (2011). A major QTL, Ghd8, plays pleiotropic roles in regulating grain productivity, plant height, and heading date in rice. Mol. Plant 4 (2), 319–330. 10.1093/mp/ssq070 21148627

[B53] Yang W. Guo Z. Huang C. Duan L. Chen G. Jiang N. (2014). Combining high-throughput phenotyping and genome-wide association studies to reveal natural genetic variation in rice. Nat. Commun. 5, 5087. 10.1038/ncomms6087 25295980 PMC4214417

[B54] Yang Y. Fu D. Zhu C. He Y. Zhang H. Liu T. (2015). The RING-finger ubiquitin ligase HAF1 mediates heading date 1 degradation during photoperiodic flowering in rice. Plant Cell. 27 (9), 2455–2468. 10.1105/tpc.15.00320 26296966 PMC4815093

[B55] Yano M. Katayose Y. Ashikari M. Yamanouchi U. Monna L. Fuse T. (2000). Hd1, a major photoperiod sensitivity quantitative trait locus in rice, is closely related to the arabidopsis flowering time gene CONSTANS. Plant Cell. 12 (12), 2473–2484. 10.1105/tpc.12.12.2473 11148291 PMC102231

[B56] Yin L. Zhang H. Tang Z. Xu J. Yin D. Zhang Z. (2021). rMVP: A memory-efficient, visualization-enhanced, and parallel-accelerated tool for genome-wide association study. Genomics Proteomics Bioinforma. 19, 619–628. 10.1016/j.gpb.2020.10.007 PMC9040015 33662620

[B57] Zhang Z. Ersoz E. Lai C. Q. Todhunter R. J. Tiwari H. K. Gore M. A. (2010). Mixed linear model approach adapted for genome-wide association studies. Nat. Genet. 42 (4), 355–360. 10.1038/ng.546 20208535 PMC2931336

[B58] Zhang Z. H. Cao L. Y. Chen J. Y. Zhang Y. X. Zhuang J. Y. Cheng S. H. (2016). Effects of Hd2 in the presence of the photoperiod-insensitive functional allele of Hd1 in rice. Biol. Open 5 (11), 1719–1726. 10.1242/bio.021071 27797723 PMC5155538

[B59] Zhao K. Tung C. W. Eizenga G. C. Wright M. H. Ali M. L. Price A. H. (2011). Genome-wide association mapping reveals a rich genetic architecture of complex traits in Oryza sativa. Nat. Commun. 2, 467. 10.1038/ncomms1467 21915109 PMC3195253

[B60] Zhao S. Jang S. Lee Y. K. Kim D.-G. Jin Z. Koh H.-J. (2020). Genetic basis of tiller dynamics of rice revealed by genome-wide association studies. Plants 9 (12), E1695. 10.3390/plants9121695 PMC7761586 33276582

