# Corrigendum: GWAS analysis to elucidate genetic composition underlying a photoperiod-insensitive rice population, North Korea

**DOI:** 10.3389/fgene.2023.1144513

**Published:** 2023-02-15

**Authors:** Chuluuntsetseg Jadamba, Richie L. Vea, Jung-Hoon Ryu, Nam-Chon Paek, Su Jang, Joong Hyoun Chin, Soo-Cheul Yoo

**Affiliations:** ^1^ Crop Molecular Breeding Laboratory, Department of Plant Life and Environmental Science, Hankyong National University, Anseong, Republic of Korea; ^2^ Bureau of Plant Industry, National Seed Quality Control Services, San Mateo, Philippines; ^3^ Department of Plant Science, Plant Genomics and Breeding Institute, Research Institute of Agriculture and Life Sciences, Seoul National University, Seoul, Republic of Korea; ^4^ Department of Integrative Biological Sciences and Industry, Sejong University, Seoul, Republic of Korea; ^5^ Carbon-Neutral Resources Research Center, Hankyong National University, Seoul, Republic of Korea

**Keywords:** GWAS, photoperiod sensitivity, heading date, North Korean rice, haplotyping, regional adaptation

In the published article, the **Funding** statement was erroneously omitted. The correct **Funding** statement appears below.

“This work was supported by the National Research Foundation of Korea (NRF) grant funded by the Korean government (MSIP) (NRF-2018R1D1A1B07050568).”

The authors apologize for this error and state that this does not change the scientific conclusion of the article in any way. The original article has been updated.

