# Supplementary figures and images for: GWAS analysis to elucidate genetic composition underlying a photoperiod-insensitive rice population, North Korea
Source: Front Genet. 2022 Dec 7;13:1036747. doi: 10.3389/fgene.2022.1036747 (PMC9768348; doi:10.3389/fgene.2022.1036747)

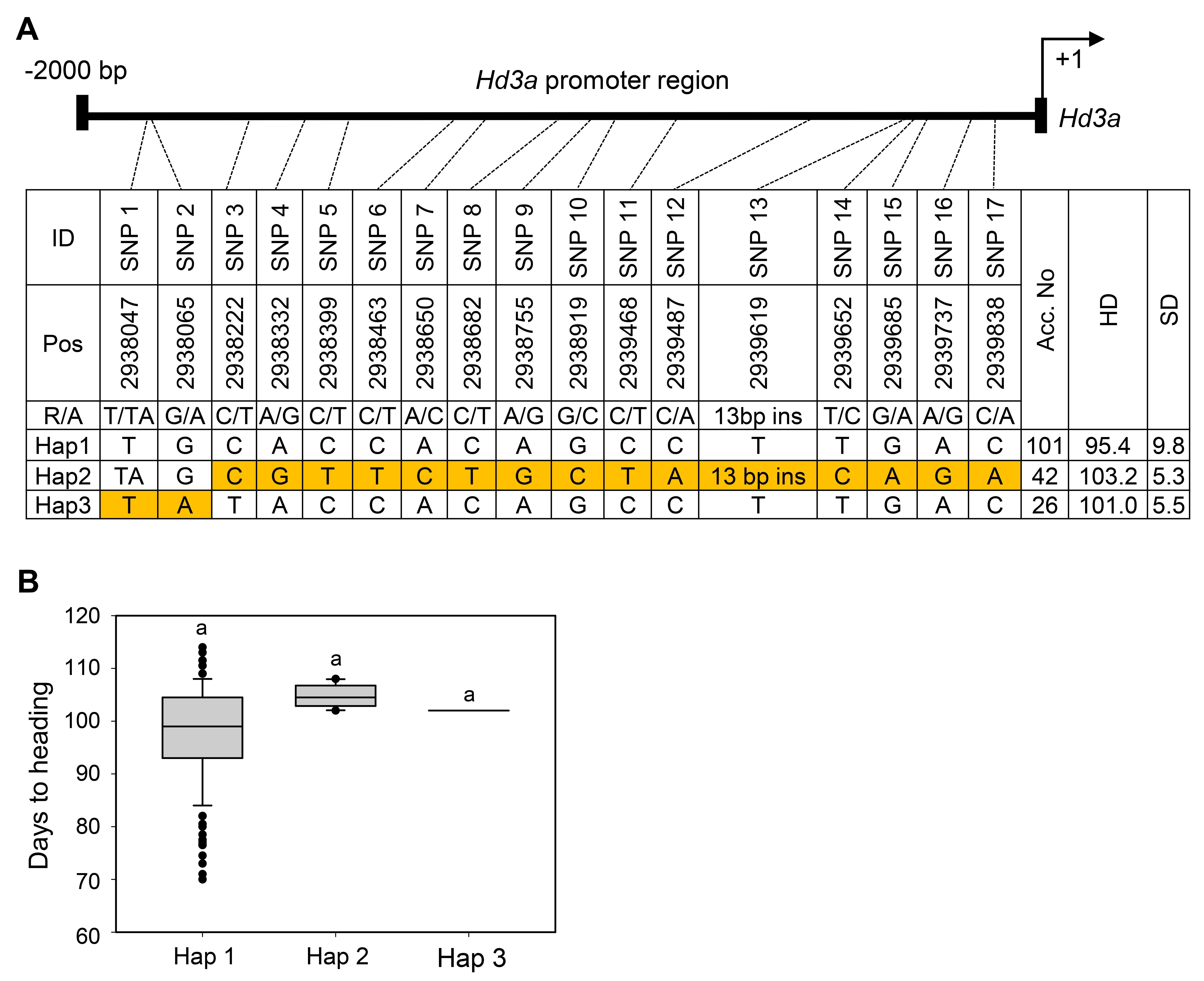

Supplement: Supplementary file 1 [file Image3.JPEG]

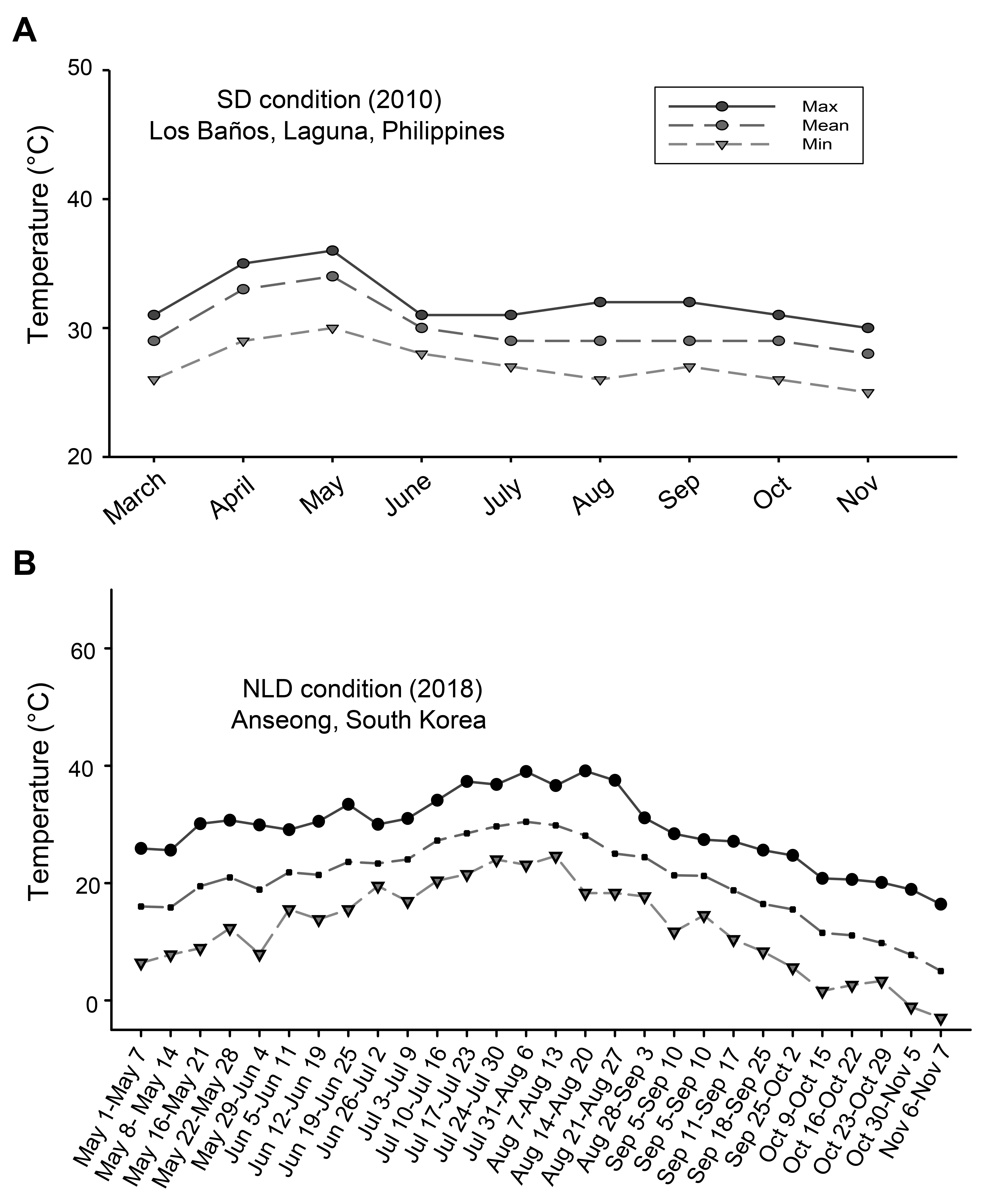

Supplement: Supplementary file 2 [file Image1.JPEG]

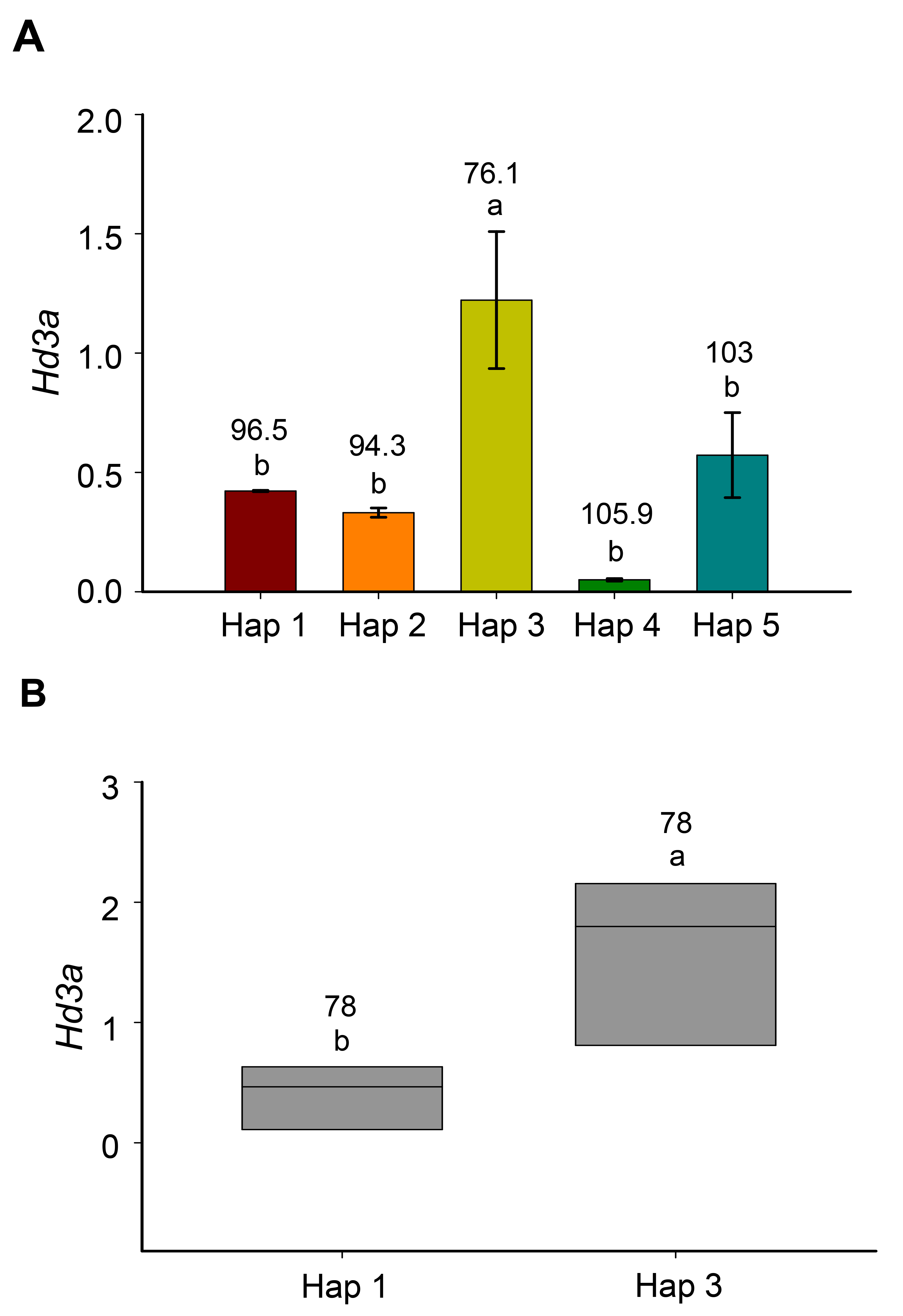

Supplement: Supplementary file 3 [file Image4.JPEG]

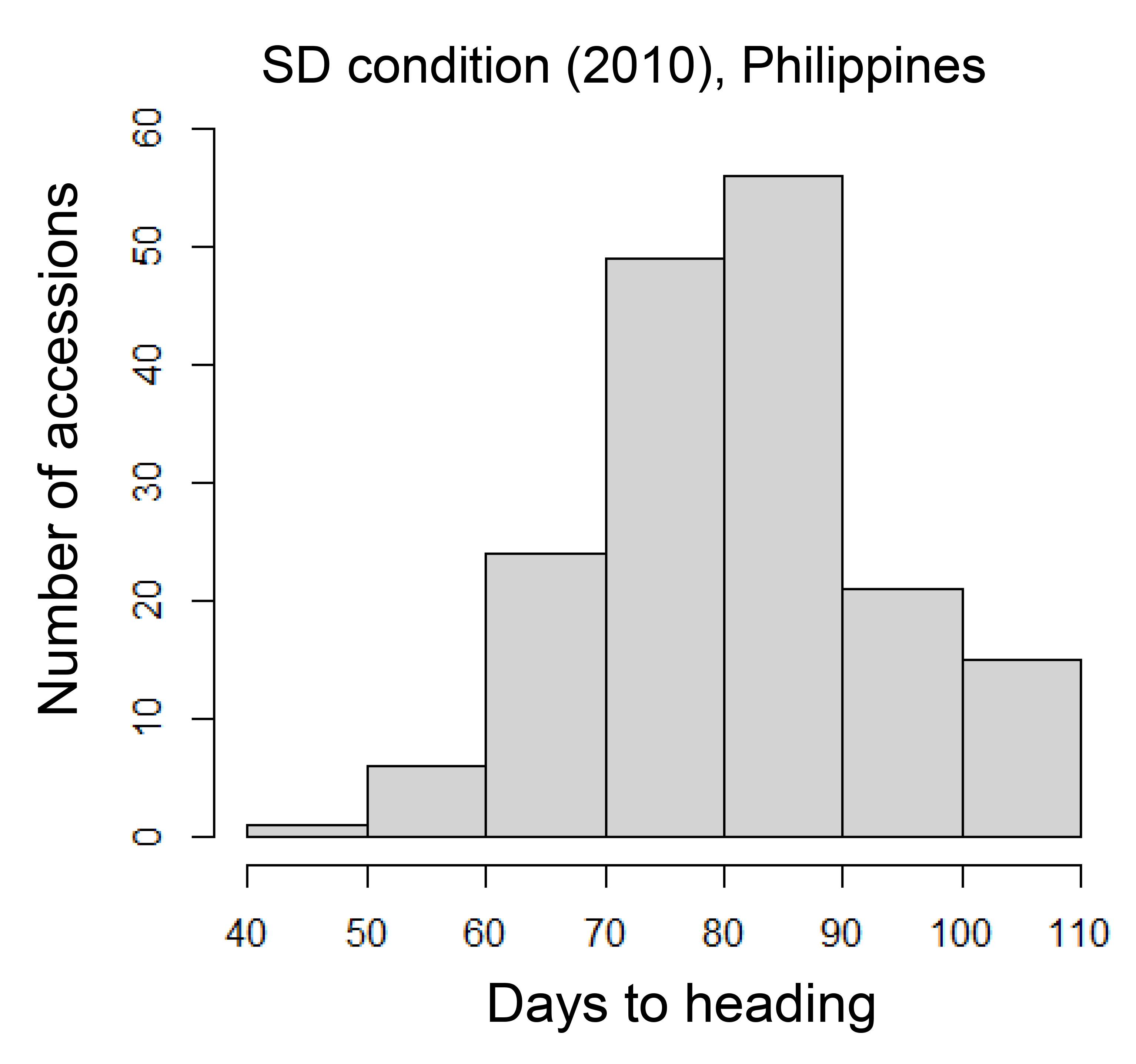

Supplement: Supplementary file 4 [file Image2.JPEG]

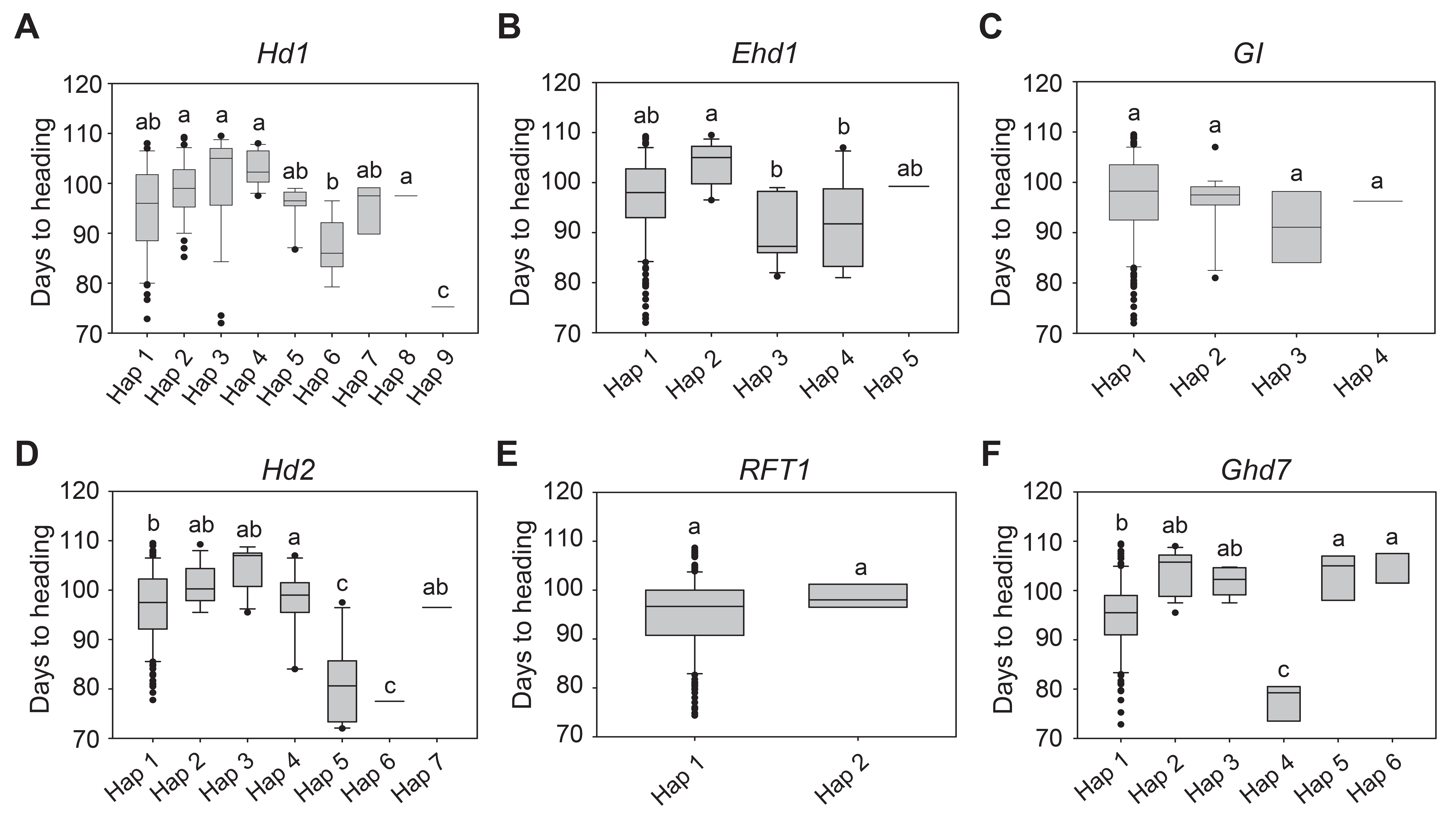

Supplement: Supplementary file 5 [file Image5.JPEG]
